# Cas9-mediated maternal effect and derived resistance alleles in a gene-drive strain of the African malaria vector mosquito, *Anopheles gambiae*

**DOI:** 10.1093/genetics/iyac055

**Published:** 2022-04-07

**Authors:** Rebeca Carballar-Lejarazú, Taylor Tushar, Thai Binh Pham, Anthony A James

**Affiliations:** Department of Microbiology & Molecular Genetics, University of California, Irvine, Irvine, CA 92697-4025, USA; Department of Microbiology & Molecular Genetics, University of California, Irvine, Irvine, CA 92697-4025, USA; Department of Molecular Biology & Biochemistry, University of California, Irvine, Irvine, CA 92697-3900, USA; Department of Microbiology & Molecular Genetics, University of California, Irvine, Irvine, CA 92697-4025, USA; Department of Molecular Biology & Biochemistry, University of California, Irvine, Irvine, CA 92697-3900, USA

**Keywords:** NHEJ, indels, gene drive, CRISPR-Cas9, resistance, mutation, mosquitoes

## Abstract

CRISPR/Cas9 technologies are important tools for the development of gene-drive systems to modify mosquito vector populations to control the transmission of pathogens that cause diseases such as malaria. However, one of the challenges for current Cas9-based drive systems is their ability to produce drive-resistant alleles resulting from insertions and deletions (indels) caused principally by nonhomologous end-joining following chromosome cleavage. Rapid increases in the frequency of such alleles may impair gene-drive dynamics. We explored the generation of indels in the germline and somatic cells in female gene-drive lineages using a series of selective crosses between a gene-drive line, AgNosCd-1, and wild-type mosquitoes. We find that potential drive-resistant mutant alleles are generated largely during embryonic development, most likely caused by deposition of the Cas9 endonuclease and guide RNAs in oocytes and resulting embryos by homozygous and hemizygous gene-drive mothers.

## Introduction

**Fig. 1.**
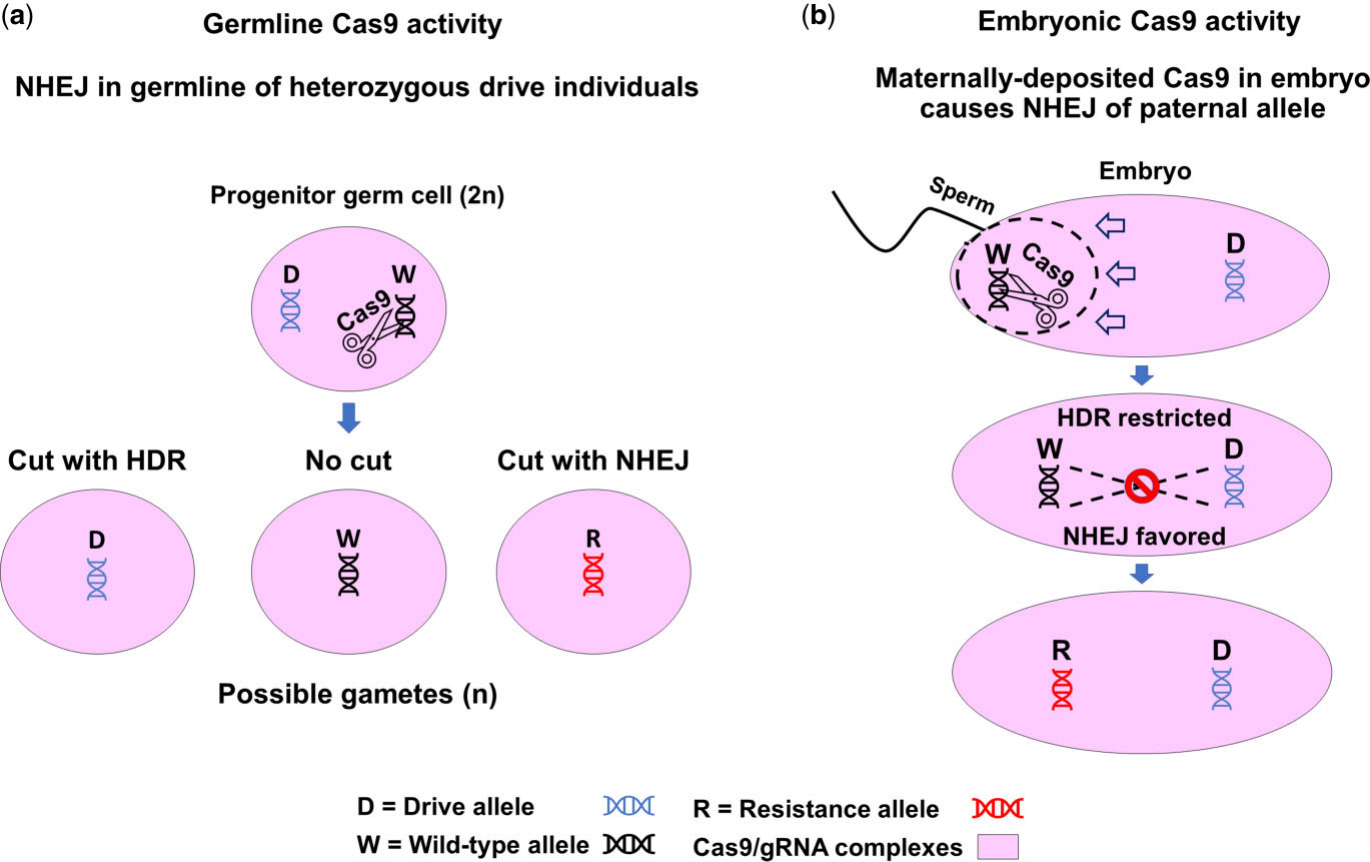
Pathways for heritable resistance allele generation. Mutant alleles that confer heritable resistance to Cas9 cleavage can result from activity of the Cas9/gRNA complexes in the progenitor germ cells and the embryos derived from eggs produced by drive-carrying females. a) The Cas9 enzyme (scissors) cleaves DNA in both male and female germline cells during the formation of gametes. Cleavage of a chromosome in a diploid progenitor germ cell prior to meiosis allows homology-directed repair (HDR) that results in conversion of a cell hemizygous for the gene-drive system to homozygosity and results in the biased inheritance of the drive system. The Cas9 enzyme may not cleave the DNA resulting in an un-altered wild-type allele or the Cas9 enzyme may cleave and the DNA repair via nonhomologous end joining (NHEJ) resulting in a potentially drive-resistant allele. Cas9 DNA cleavage in postmeiotic germ cells also would result in NHEJ as there would be no template for HDR. b) Cas9/gRNA complexes may be deposited in the female germ cells and as a result, be present in the resulting embryo following fertilization. These maternally-inherited complexes in the embryo can act on the incoming paternally- donated allele following its release from the sperm head. If the paternally-donated allele is cleaved just after release of the male pronucleus, the physical distance between the paternal and maternal chromosomes within the embryo may prevent the female chromosome serving as a template for HDR resulting in NHEJ and a potentially drive-resistant allele. Furthermore, perdurance of the Cas9/gRNA complexes in somatic cells may produce mutations that lead to mosaicism following blastoderm formation and the observed mosaic “tear” eye phenotype.

CRISPR/Cas9-based gene drives are promising and powerful novel tools for controlling vector-borne diseases due to their ability to bias the inheritance of beneficial synthetic genes and preferred alleles during vector insect reproduction (Deredec *et al.* 2008; Champer *et al.* 2016; Gantz and Bier 2016; Godfray *et al.* 2017). Two approaches currently take advantage of gene-drive systems: population modification and population suppression (Gantz *et al.* 2015; Hammond *et al.* 2016; Kyrou *et al.* 2018; Adolfi *et al.* 2020; Carballar-Lejarazú *et al.* 2020; Simoni *et al.* 2020). Proofs-of-principle for both approaches have been demonstrated in malaria-vectoring mosquito species, and they have high-efficiency drive and are able to spread genes-of-interest into caged laboratory populations in as few as 4 generations when releases of gene drive-carrying males were performed at ratios of 1:1 with wild-type competitors (Kyrou *et al.* 2018; Adolfi *et al.* 2020; Carballar-Lejarazú *et al.* 2020; Simoni *et al.* 2020). One potential challenge to these gene-drive systems is the generation of guide RNA (gRNA) target-site mutant alleles. There are 2 potential pathways that can create mutant alleles in a Cas9-based gene-drive system (Fig. 1). Mutant alleles can be formed in the germline of an individual carrying 1 copy (hemizygous for the insertion) of the drive system if Cas9/RNA-mediated DNA cleavage does not result in homology directed repair (HDR) (and drive) and a nonhomologous end-joining (NHEJ) event occurs during repair of the lesion. Mutant alleles also can occur due to Cas9/gRNA activity if the complexes are in cells other than the developing germ-line. These somatic Cas9/gRNA complexes may be present if the activity of the promoter driving Cas9 expression is not restricted fully to germline tissues, or could result from maternally-deposited complexes in the oocyte and embryo that cleave the paternally-donated wild-type allele (Gantz *et al.* 2015; Champer *et al.* 2017). This somatic activity can result in a mosaic eye-color phenotype comprising wild-type and mutant ommatidia (Gantz *et al.* 2015; Carballar-Lejarazú *et al.* 2020). The NHEJ mutant alleles created from these pathways may be resistant to subsequent gRNA-directed Cas9 cleavage and thereby reduce or inhibit introduction of the desired genetic traits if the mutant alleles are passed down to future generations (Hammond *et al.* 2017; Pham *et al.* 2019).

We designed and generated a gene-drive line, AgNosCd-1, for population modification of the African malaria mosquito, *Anopheles gambiae*, that has a Cas9/gRNA-based autonomous gene-drive system marked with the cyan fluorescent protein (CFP). The drive system targets the second chromosome, autosomal *cardinal* (*cd*) gene ortholog (*Agcd*, AGAP003502; VectorBase;  Supplementary Table 1) producing a recessive red-eye phenotype in individuals that have both copies of the gene disrupted (Carballar-Lejarazú *et al.* 2020). AgNosCd-1 achieves drive efficiencies of 98–100% in males and females with a small proportion of the progeny from females exhibiting mosaic eyes (<9%) or loss-of-function phenotypes (<2%) (Carballar-Lejarazú *et al.* 2020). While the results of small cage trial experiments showed that AgNosCd-1 was not inhibited by the accumulation of drive-resistant alleles, further evaluation of resistant allele formation and impact on gene-drive efficiency is needed to enhance modeling and predictions in anticipation of field-release studies. We examined the generation, inheritance patterns, sequence diversity, and stability of potential drive-resistant gRNA target-site alleles during crosses of AgNosCd-1 individuals to wild-type and mutant *cd* mosquitoes.

## Materials and methods

### Mosquito strains and maintenance

Colonies of the *An.*  *gambiae* G3 strain (referred hereafter as wild-type, WT), the *An.*  *gambiae* G3 X1 strain (referred hereafter as X1 wild-type, X1-WT) (Volohonsky *et al.* 2015), the transgenic lines AgNosCd-1 (Carballar-Lejarazú *et al.* 2020) and AgTP13 (Carballar-Lejarazú *et al.* in preparation) expressing CFP in the eyes, and 2 strains, Ag*cd*^Δ*11,14*^ and Ag*cd*^Δ^*^11^*, with potential loss-of-function mutations in the *Agcd* gene ortholog (abbreviated hereafter as *cd*), are the sources of all insects used in this experiment. The AgTP13 line couples the AgNosCd-1 gene-drive system (carrying the 3xP3 promoter driving the CFP as a dominant marker) to a pair of engineered genes encoding the mosquito codon bias-optimized murine single-chain antibodies (scFvs), m1C3 and m2A10, targeting *Plasmodium falciparum* ookinetes and sporozoites, respectively (Isaacs *et al.* 2011). A homozygous *cd* mutant line, Ag*cd*^Δ*11,14*^, was established by outcrossing a male mosquito with the *cd* red-eye phenotype and no CFP fluorescence (*cd*^-^/CFP^−^) recovered from an AgNosCd-1 cage trial (Carballar-Lejarazú *et al.* 2020) to 15 hemizygous AgNosCd-1 females. The *cd*^-^/CFP^+^ progeny were subsequently intercrossed to generate homozygous *cd*^-^/CFP^−^ mosquitoes in the next generation. Molecular analysis of the target site and sequencing detected 2 deletions of 11 (*cd*^Δ*11*^) or 14 (*cd*^Δ*14*^) nucleotides (nt) in length. Subsequent *cd*^-^/CFP^−^ intercrosses and molecular screening generated the homozygous Ag*cd*^Δ*11*^ line, confirming that this deletion alone imposes a loss-of-function phenotype.

All mosquitoes were maintained at 27°C with 77% humidity and a 12-h day/night, 30 min dusk/dawn lighting cycle. Adult females were provided rabbit blood (Colorado Serum Company, CO) through artificial membranes (Hemotek, Inc., Blackburn, UK).

### Mosquito mating and screening

Mosquitoes were mated in pools with the number of males and females in each pool determined by their availability as the number of each unique phenotype generated by 1 cross and used in a subsequent cross could not be predetermined. Mating events of ∼100 individuals were performed in 83-ounce (2455 cm^3^) cages and matings of <100 individuals in 46-ounce (1360 cm^3^) cages. Males and females in each cross were allowed to mate for 5 days, then provided a blood meal and oviposition cup 3 days post blood meal. The progeny of each cross were screened using light microscopy and UV-fluorescence for eye phenotypes (eye color: red, *cd* mutant; black, wild-type) and the presence or absence of CFP fluorescence, and hand-sorted as pupae by sex. Pupae were kept separately in 16-ounce (473 cm^3^) cups by sex and eye phenotypes and subsequent emerging adults were maintained as virgins. Individuals of each unique phenotype were used either in subsequent mating events or preserved at −20°C for molecular analyses.

### Crosses to generate potential drive-resistant alleles

Maternal deposition of Cas9 and gRNAs into eggs can cause mutations following Cas9-induced, nonhomologous end-joining (NHEJ) resulting in indel mutations at the gRNA target site of the male-contributed allele (Gantz *et al.* 2015). We outcrossed separately 50 hemizygous or homozygous AgNosCd-1 females to 50 WT males in triplicate and screened the progeny for the red-eye phenotype that would indicate that an incoming wild-type *cd* allele from the males had been mutated. We refer to mosquitoes as hemizygous or homozygous based on whether they carry 1 or 2 copies of the drive system, respectively.

### Crosses to assess mutant *cd* allele inheritance

#### Mutant *cd* allele/AgNosCd-1 hemizygote intercrosses

A total of 47 progeny with a red-eye color and CFP^+^ phenotype generated from a homozygous AgNosCd-1 female outcrossed to WT males were presumed to have 1 copy of the drive allele and 1 copy of a nonfunctional mutant *cd* allele or some level of mosaicism not apparent in the eye for indel and wild-type alleles. All individuals with this phenotype were combined and allowed to intercross and the progeny screened again for red-eyes and CFP fluorescence. The red-eye/CFP^−^ mosquitoes were presumed to have 1 (homozygous) or 2 heteroallelic nonfunctional mutations in the *cd* gene.

#### Mutant *cd* allele/AgNosCd-1 hemizygote outcrosses

A total of 15 red-eye*/*CFP^+^ hemizygous males were removed from the previous hemizygote intercross cages after their mating period, allocated to a new cage and outcrossed to 75 WT females. All progeny were screened for the red-eye phenotype and CFP fluorescence.

#### Mutant *cd* allele homozygote intercross

Approximately 60 mixed-sex progeny generated from the previous hemizygote intercross with evidence of 2 copies of nonfunctional mutant *cd* alleles based on the red-eye, CFP^−^ phenotype were combined and allowed to intercross. All progeny were screened for the red-eye and CFP fluorescence phenotypes.

### Molecular analysis of individual mosquitoes from crosses

Genomic DNA was extracted from individual mosquitoes using either a Promega Wizard Genomic DNA Purification Kit or Extract-N-Amp PCR kit (Sigma). Two oligonucleotide primers (5′GTACTCGTACGGTCGCTCCTTA3′ and 5′ATTGTTGTTGCAGATGAGTCGT3′) were designed to amplify a DNA fragment of 396 base pairs (bp) in length encompassing the gRNA-directed Cas9 cleavage site. Two additional primers (5′CTCCAGCAGCTCCTTATCGC3′ and 5′GCTTGTTTGAATTGAATTGTCGC3′) were used to amplify a 742 bp DNA fragment spanning the *cd* gene left homology arm and the drive cassette to verify the presence of the drive allele. Sanger sequencing was used to analyze potential indels in genomic DNA from mosquitoes recovered from each mating event (Genewiz, USA).

### Mutant *cd* allele dynamics in a gene-drive population

An 83-ounce (2455 cm^3^) cage was set up with 50 red-eye/CFP^−^ males from the *cd* homozygous mutant line Ag(*cd*  ^Δ*11,14*^) containing a mixture of mutant *cd* alleles, *cd*^Δ*11*^ or *cd*^Δ*14*^, and 50 AgTP13 gene-drive homozygous females. Progeny were screened for eye color and CFP fluorescence, and 300 pupae were selected randomly to populate the next generation. The population was maintained in this manner for 8 generations to assess the stability over time of mutant *cd* and drive alleles in a mixed population.

### *cd* ^Δ*11*^ allele dynamics in wild-type populations

Triplicate 83-ounce (2455 cm^3^) cages were established with 150 homozygous Ag*cd*^Δ^*^11^* males, which contained only the 11 nucleotide deletion, and 150 X1-WT females. All progeny were screened for eye color and fluorescence phenotypes, 300 pupae selected randomly and transferred into a new cage for the next generation. The cage trials were maintained for 10 generations to determine the dynamics of the *cd*^Δ^*^11^* alleles in the population over time.

## Results

### Assessment of NHEJ-induced indel allele formation in embryos from AgNosCd-1 females

**Fig. 2.**
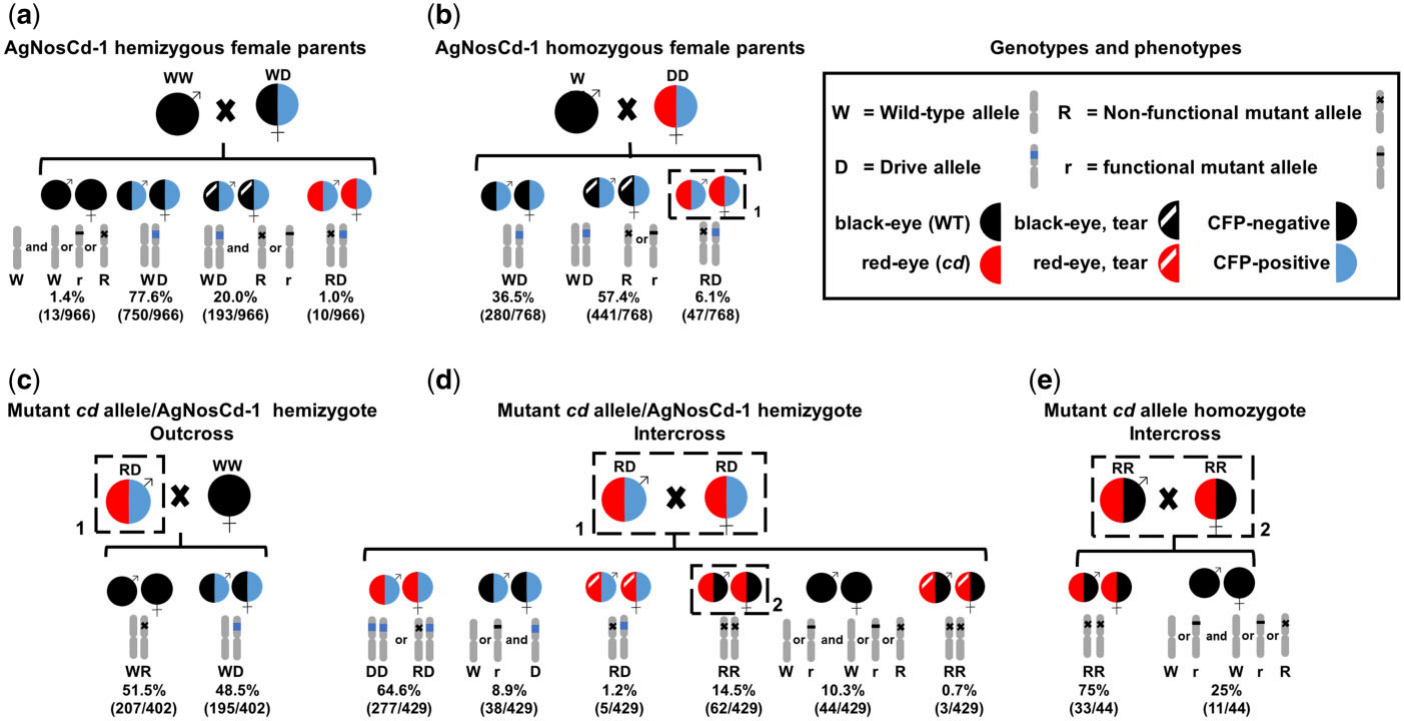
Crosses to generate potential drive-resistant *cd* mutant alleles. Crosses were made with homo- or hemizygous AgNosCd-1 (CFP^+^) males and females to wild-type (outcrosses) or sibling (intercrosses) mosquitoes to detect and monitor the formation and inheritance patterns of mutant *cd* alleles. Graphic representations of parental and progeny phenotypes (half-circle coloration) and alternative genotypes (marked chromosome symbols) are presented along with phenotypic frequencies. The left half of each circle denotes the eye color phenotype of wild-type (black), *cd* mutant (red) or mosaic, tear (white slash) in a wild type- or *cd*-mutant background (black or red, respectively). The right half of the circle denotes negative or positive for CFP fluorescence (black or blue, respectively). The numbers of each phenotype seen and total number of mosquitoes scored are shown in parentheses. Top panels: crosses used to generate mutant *cd* alleles, a) hemizygous and b) homozygous female CFP^+^ individuals were used to increase the likelihood of recovery of NHEJ-induced mutant *cd* alleles resulting from maternal effects as seen in Carballar-Lejarazú *et al.* (2020), with 50 AgNosCd-1 (hemizygous or homozygous) females crossed with 50 wild-type (WT) males in triplicate. Individual experimental details are presented in Supplementary Tables 2 and 3. Bottom panels c and d: crosses with selected progeny obtained in b (dashed box outline labeled “1”) with potential mutant *cd* alleles to assess the inheritance patterns of mutant *cd* alleles in the presence or absence of a chromosome carrying the drive system. c) A total of 15 red-eye*/*CFP^+^ hemizygous males were removed from the hemizygote intercross cage (Cross D) after their mating period, allocated to a new cage and outcrossed to 75 WT females. d) A total of 47 female and male red-eye/CFP^+^ homozygotes generated in b were intercrossed. e) Phenotypes and possible genotypes of progeny from an intercross of ∼60 mixed-sex red-eye/CFP^−^ progeny from d (dashed box outline labeled “2”).

Separate outcrosses of homozygous and hemizygous AgNosCd-1 females to WT males were used to determine the frequencies of NHEJ-induced indel allele formation due to Cas9 deposition in embryos (Fig. 2, a and b). Next-generation progeny with a red-eye, CFP positive (*cd^-^*/CFP^+^) phenotype were likely to have a nonfunctional indel allele formed during early embryonic development. These individuals would have the drive allele (CFP^+^) inherited from their mothers and 1 mutant *cd* allele resulting from mutation of the paternally derived gene. The frequency of recovery of red-eye phenotypes was significantly higher in progeny resulting from outcrosses of homozygous AgNosCd-1 females (6.1% red-eye/CFP^+^) compared to hemizygous AgNosCd-1 outcrosses (1.0% red-eye/CFP^+^) (2 proportion *z*-test, *z* = 5.94; *P* ≤ 0.00001) (Fig. 2, a and b; Supplementary Tables 2 and 3). Sequencing around the gRNA target site of samples of *cd^-^*/CFP^+^ progeny from both homozygous and hemizygous AgNosCd-1 females (18 and 4 individuals, respectively) identified 29 distinct indels, with 1 mutation appearing in individuals (10 and 20, Supplementary Table 4) recovered from both crosses. These data support the conclusion that the indels occurred in the fertilized embryo as they all were different, which would not be expected if the progeny shared a mutant allele inherited from either parent. Eight red-eye/CFP^+^ individuals (2, 4, 8, 9, 14, 17, 19, and 22) had 2 different mutant *cd* alleles along with the drive allele, indicating that multiple indels were generated during embryonic development resulting in a mosaic mosquito. Six indels (7, 9b, 12, 13, 14a, and 17b) were in frame, but the size or position of the indels affected the function of the resulting product and failed to restore the wild-type phenotype. We also observed a greater portion of phenotypically mosaic individuals (termed “tear eye”; Carballar-Lejarazú *et al.* 2020) in the progeny of homozygous AgNosCd-1 outcrosses (57.4% tear-eye/CFP^+^) compared to hemizygous AgNosCd-1 outcrosses (20.0% tear-eye/CFP^+^) (*z* = 16.06; *P* *< *0.00001) (Fig. 2, a and b). The high frequency of recovery of mosaic, tear-eye phenotype mosquitoes is consistent with most embryonic Cas9 cleavage of the incoming wild-type male *cd* allele occurring later in development, after several mitotic divisions, which gives rise to mutant alleles in some, but not all, somatic cells (Carballar-Lejarazú *et al.* 2020, Fig. 1).

### Assessment of functional mutant *cd* allele formation in AgNosCd-1 female germlines

Progeny negative for the gene-drive allele with a wild-type, black eye color (black-eye/CFP^−^) resulting from outcrosses of hemizygous AgNosCd-1 females to WT males may have different types of allele combinations. They may have 2 wild-type *cd* alleles, one from their father and the second as a result of unsuccessful germline drive conversion in their hemizygous mothers. They may have 1 paternally-inherited wild-type allele and a maternally-inherited mutant *cd* allele due to NHEJ following unsuccessful germline drive conversion. They also may have a wild-type allele from the mother due to lack of conversion and a functional or nonfunctional allele from the male *cd* locus mutated by the maternal deposition of Cas9 in the embryo. There were no black-eye/CFP^−^ progeny recovered from outcrosses of homozygous AgNosCd-1 females to wild-type males because both of the parental female *cd* genes have insertions of the gene-drive construct (Fig. 2b). However, 1.4% (13/966) black-eye/CFP^−^ progeny were recovered from outcrosses of hemizygous AgNosCd-1 females to wild-type males (Fig. 2a). Molecular analysis was done to examine if these individuals were homozygous or heterozygous for wild-type alleles to estimate the rate of formation of resistance allele in the germline. All 13 black-eye/CFP^−^ individuals were homozygous for WT alleles indicating that no functional mutant alleles were generated in the AgNosCd-1 hemizygous female germline with the sample size used here (Supplementary Table 5).

### Inheritance of nonfunctional mutant *cd* alleles

Mutant *cd* allele-bearing individuals were mated to assess how the alleles are inherited in the presence or absence of gene-drive alleles. Two crosses with mutant *cd* allele and drive allele hemizygotes, red-eye/CFP^+^, and 1 cross with *cd* mutant allele homozygotes, red-eye/CFP^−^, were performed.

#### Mutant *cd* allele/AgNosCd-1 hemizygote outcrosses

Males hemizygous for mutant *cd* (red-eye) and gene-drive (CFP^+^) alleles were outcrossed to WT females to determine whether the drive allele could convert *cd* mutant alleles in the male germline (Fig. 2c). The mutant *cd* alleles represent a number of different genotypes in individual male progeny resulting from the cross depicted in Fig. 2b. Segregation of the gene-drive allele was consistent with Mendelian inheritance with the CFP^+^ and CFP^−^ phenotypes seen in 48.5% (195 of 402) and 51.5% (207 of 402), respectively (*Χ*^2^ = 0.179, *P* *= *0.67), of the progeny. This is consistent with no gene-drive conversion in the germline of the AgNosCd-1 hemizygote males, and we interpret the mutant target *cd* alleles to be drive-resistant.

#### Mutant *cd* allele/AgNosCD-1 hemizygote intercrosses

Intercrosses of hemizygous gene-drive and mutant *cd* males and females were done and the resulting CFP^+^:CFP^−^ phenotypic ratio in the progeny was 74.6% (320/429):25.4% (109/429), consistent with a canonical 3:1 Mendelian phenotypic inheritance pattern (*Χ*^2^ = 0.038, *P* *= *0.845; Fig. 2d). Again, these data support the interpretation that the mutant *cd* target alleles are resistant to the gene-drive system.

The distribution of eye-color phenotypes among the progeny of the hemizygous intercross reflects the impact of the maternal deposition of the Cas9 protein during early development. While this was an intercross of parents hemizygous for the gene-drive construct as evidenced by all parents being CFP^+^, the other allele was expected to be a nonfunctional mutant *cd* allele as all parents also had the red-eye color phenotype. However, 6 eye-color phenotypes were recovered: red-eye/CFP^+^, red-eye/CFP^−^, black-eye/CFP^+^, black-eye/CFP^−^, mosaic “tear”/CFP^+^ and mosaic “tear”/CFP^−^ (Fig. 2d). In addition, a rare mosaic phenotype in an apparent mutant *cd*, red-eye background was seen, most likely reflecting a heteroallelic combination of a null and hypomorph mutation (Supplementary Fig. 1). Molecular analyses showed that the genotypes of these individuals can explain the observed phenotypes and provided more insight into the diversity of drive-resistant, mutant *cd* alleles created in AgNosCd-1 female-derived embryos (Supplementary Table 6). The 10 red-eye/CFP^+^ progeny were either homozygous for the drive alleles (6) or hemizygous with 1 drive allele and 1 out-of-frame mutant allele (4). This is expected based on the assumption that their hemizygous parents contributed either an AgNosCd-1 allele or a nonfunctional mutant *cd* allele. The 9 black-eye/CFP^+^ phenotypic individuals were not expected because the red-eye hemizygous parents should not have been able to provide a functional *cardinal* gene. However, the genotypes of these progeny revealed them to be hemizygous for the drive allele and 1 of 2 versions of a functional resistant allele with small (6 nt) in-frame mutations. The 12 black-eye/CFP^−^ individuals each had 1 of 6 heteroallelic genotypes characterized by 1 out-of-frame and 1 small in-frame mutant *cd* allele. The 10 red-eye/CFP^−^ individuals were an expected phenotype but they had an unexpected diversity of alleles with individuals with either 2 out-of-frame mutant *cd* alleles or 1 out-of-frame allele along with an allele with large in-frame mutation resulting from 5 heteroallelic genotypes and 10 total alternate alleles.

Comparisons of the DNA sequences of the alleles in the black-eye progeny (Supplementary Table 6, derived from the cross depicted in Fig. 2d) and a subset of their mutant *cd* red-eye/CFP^+^ parents (Sequences 1–18, Supplementary Table 4) support the conclusion that some of the parents were genotypically mosaic. The black-eyed progeny likely resulted from mutant red-eye/CFP^+^ parents that carried 2 different *cd* mutant alleles in their germline along with the drive allele. The in-frame mutant *cd* alleles were transmitted subsequently to the progeny and restored the wild-type, black-eye phenotype. The mutant red-eye color originates in a clone or clones of cells that formed the ommatidia and had nonfunctional *cd* mutant alleles. The recovery at low frequency of the mosaic “tear”-eye phenotypes is consistent with this interpretation (Fig. 2d).

#### Mutant *cd* allele homozygous intercrosses

An intercross of the small number (14) of red-eye/CFP^−^ (drive system-negative) mosquitoes originating from the hemizygous intercross (Fig. 2d) was made to further understand the inheritance of the resistant alleles (Fig. 2e). The expected result of an intercross of red-eye/CFP^−^ individuals, with presumably 2 nonfunctional mutated alleles, is 100% of the offspring with 2 nonfunctional alleles, sharing the red-eye/CFP^−^ phenotype of their parents. However, this intercross again produced an unexpected outcome as offspring with 2 distinct phenotypes, red-eye/CFP^−^ and black-eye/CFP^-^, were recovered. Sequencing data revealed that the red-eye/CFP^−^ individuals carried as expected 2 different out-of-frame mutated *cd* alleles (1–5.a , 1–5.b) (Supplementary Table 7). Seven of the 8 black-eye/CFP^−^ individuals (2–8) sequenced were hemizygous with 1 out-of-frame resistant allele and 2 different in-frame resistant alleles (2–8.b and 2–8.c) that had the large insertion fragment coming from a region downstream of the target site, resulting in duplication of part of the gene that restored function. One individual had both an in-frame and out-of-frame allele (1.a, 1.b).

Calculating the rate of formation of heritable, functional potentially drive-resistant alleles generated by NHEJ is complicated by phenotypic and genotypic mosaicism, cluster effects from events in single germline progenitor cells and the ability to sequence the target sites of large numbers of mosquitoes. The difficulty is demonstrated from the work from the B and D crosses in Fig. 2. The frequency of mosaic, tear eye/CFP^+^ (57.4%) and red-eye/CFP^+^ (6.1%) progeny was high (63.5%) from a cross of wild-type males with presumed homozygous gene-drive mothers (red eye/CFP^+^) indicating a high rate of NHEJ in the embryos of those females (Fig. 2b). Recall that a functional resistance allele would be expected to produce a dominant wild-type (black-eye) phenotype and none of the red-eye/CFP^+^ have that. However, an intercross of the red-eye/CFP^+^ progeny from this cross produced 8.9% black-eye/CFP^+^ and 10.3% black-eye/CFP^−^ progeny for a combined total of 19.2% black-eye mosquitoes, presumably carrying one or more functional NHEJ alleles inherited from their red-eye/CFP^+^ mothers (Fig. 2d). These females must have been mosaic in their germlines for black-eye conferring alleles. The sequences of the DNA surrounding the target sites of 19 of these mosquitoes revealed 14 distinct NHEJ indel alleles, 8 of which were in-frame as would be needed to provide the black-eye phenotype. All 9 of the 8.9% black-eye/CFP^+^ progeny were hemizygous for the gene-drive construct and a functional allele, as expected. The 10 black-eye/CFP^−^ progeny showed 2 apparent clusters (identical genotypes, 1–4 and 6–7, Supplementary Table 6). The challenge then becomes trying to define the denominator. We sampled by subsequent mating only a small fraction of the progeny (14 total red eye/CFP^+^) of the original cross (Fig. 2b) and it can be expected that the whole cohort is likely to have produced more than the 14 distinct alleles. The best way to get an accurate estimate would be to sequence a large number of the progeny of the cross in Fig. 2b and use those results to estimate the frequency.

### Population dynamics of the homozygous Ag*cd*^Δ11,14^ line in cage population competition with a gene drive line

A cage population trial initiated with 50 heteroallelic Ag*cd*^Δ^*^11,14^* [*cd* mutant line containing 2 different mutations, an 11-nucleotide-deletion *cd*^Δ*11*^and a 14-nulceotide-deletion, *cd*^Δ*14*^ (Supplementary Table 8)] males and 50 homozygous AgTP13 gene-drive females was performed to evaluate how a potential drive-resistant mutant *cd* allele might affect gene-drive dynamics over multiple generations. AgTP13 couples the autonomous AgNosCd-1 gene-drive system to 2 antimalarial single-chain antibodies, m1C3 and m2A10, driven by the carboxypeptidase-encoding and vitellogenin-encoding gene promoters, respectively (Carballar-Lejarazú *et al.* in preparation).

**Fig. 3.**
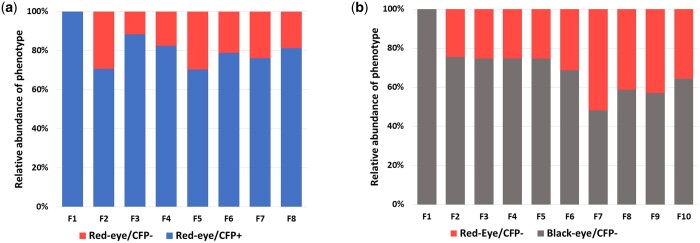
Population dynamic of mutant, drive resistant line Ag*cd*^Δ*11,14*^ in the presence of drive or wild-type (WT) alleles. a) Male homozygous red-eyed Ag*cd*^Δ*11,14*^ mutant mosquitoes were crossed with AgTP13 (red-eyed/CFP^+^) females. b) Triplicate crosses between homozygous red-eyed mutant Ag*cd*^Δ^*^11^* male and *Anopheles gambiae* X1 females were performed. Averaged data for each generation are presented here. Individual experimental details are presented in Supplementary Table 10. Progeny from both a and b were screened and the percentages recorded of the eye color (*cd*, red-eye or WT, black-eye) and fluorescence (CFP^+^ or CFP^−^) phenotypes. Progeny were allowed to intercross for subsequent generations.

The first generation (F1) progeny are expected to be hemizygous for *cd*^Δ^*^11,14^* and AgTP13 drive alleles and therefore be 100% red-eye/CFP^+^. This expectation was verified in all the F1 progeny (Fig. 3a, Supplementary Table 9). However, a small number of progeny recovered in the first generation had only the *cd*^Δ*11*^ allele being represented in subsequent generations (see *Materials and*  *Methods*: “Mutant *cd* allele dynamics in a gene-drive population”). In addition, it was anticipated that *cd*^Δ^*^11^,* if a true resistance allele, would inhibit AgTP13-mediated target-site conversion in subsequent interbreeding populations (F2–F8) resulting in phenotypic ratios of ∼3:1 CFP^+^:CFP^−^ as the hemizygous mosquitoes continue to intermate. The percentages of CFP^+^ drive-carrying mosquitoes fluctuated between 71% and 88%, with the 2 generations (F3, F4) above 82% being statistically significant (Pearson’s *Χ*^2^ test with Bonferroni correction, *Χ*^2^ ≥ 23.5, *P* ≤ 8.4 × 10^−6^). While there is a failure of AgTP13 to achieve full introduction (every mosquito having at least 1 copy of the gene-drive construct), thus verifying that Ag*cd*^Δ^*^11^* is a true drive-resistant line, there appears to be no major load on the drive allele and it can be maintained in the population even in the presence of the resistance allele. The fluctuations in the inheritance bias may reflect stochastic properties of the cage trial design or possible low-frequency cleavage of the resistant *cd*^Δ^*^11^* allele.

### Population dynamics of the homozygous AgcdΔ11 line in cage population competition with WT

Ag*cd*^Δ*11*^males were crossed in the presence of equal numbers of wild-type X1-WT females to assess their competitive success over time at contributing to subsequent generations in a wild-type population. The first generation (F1) progeny of these crosses was heterozygous for 1 wild-type and 1 *cd*^Δ^*^11^* allele and show the black-eye phenotype (Fig. 3b, Supplementary Table 10). Assuming that there is no competitive disadvantage associated with the *cd*^Δ^*^11^* mutant allele, it is expected that all subsequent generations would maintain an ∼3:1 ratio of wild-type black-eye to *cd*^Δ^*^11^* red-eye phenotypes as the populations continue to interbreed. No significant deviations from this ratio were observed for the next 4 (F2–F5) generations (χ^2^ ≤ 0.054, *P* > 0.8). However, at the F6 and subsequent generations (F7–F10), the percentages of homozygous *cd*^Δ^*^11^* mutant progeny increased and fluctuate between 30% and 50%. This increase and fluctuation varied greatly among the 3 experimental replicates (Supplementary Table 10). These data support the conclusion that the *cd*^Δ^*^11^* nonfunctional resistant allele does not carry a greater load than wild-type alleles and the red-eye phenotype is not selected against in a mixed population.

## Discussion

AgNosCd-1 hemizygote female lineages were shown previously to have a high drive efficiency with the majority of progeny (∼95%) from AgNosCd-1 female outcrosses to wild-type mosquitoes exhibiting the gene-drive (CFP^+^) phenotype (Carballar-Lejarazú *et al.* 2020). This high-drive efficiency was attributed in part to low rates of resistance allele formation as a consequence of NHEJ. However, it also was observed that 2 copies of a Cas9-containing allele can increase the rates of resistance allele formation, presumably due to increased levels of Cas9-mediated cleavage and subsequent NHEJ in embryos (Gantz *et al.* 2015; Champer *et al.* 2017, 2018). Although homozygous AgNosCd-1 females had a higher embryonic NHEJ allele generation rate compared to hemizygous AgNosCd-1 (6.1% and 1.4%, respectively), they are lower than what has been observed with similar crosses in other Cas9-based gene-drive systems in *Anopheles*  *stephensi* (estimated at 65%–90%) or *Drosophila melanogaster* (estimated between 5% and 100% dependent on drive and genetic background) (Gantz *et al.* 2015; Champer *et al.* 2017; 2018; 2019). The lower rate of embryonic NHEJ allele generation might be in part attributed to the differences among drive systems in the control DNA used to express the Cas9 nuclease. AgNosCd-1 utilizes the *An. gambiae nanos* ortholog regulatory DNA that may better localize Cas9 activity to the germline cells, unlike other promoters such as *vasa*, which can cause the expression of Cas9 outside of the germline (Terradas *et al.* 2021).

The progeny of crosses affected by embryonic Cas9 activity produced NHEJ alleles that could inhibit gene-drive. Mutant *cd*^Δ^*^11^* alleles appear to not be converted (or converted at a low rate, Fig. 3a) in the germline of mosquitoes hemizygous for the AgNosCd-1 or AgTP13 drive systems, and they suppress the significant biased inheritance in both intercrosses and outcrosses to wild-type mosquitoes.

A significant observation from these crosses and subsequent sequencing of the progeny was the observation that the red-eyed/CFP^+^ mutant *cd* allele/AgNosCd-1 hemizygous individuals generated from embryonic Cas9 activity were mosaic. The DNA sequences of mutant *cd* allele/AgNosCd-1 hemizygous individuals revealed some individuals with 2 different mutant *cd* alleles in addition to the drive allele. Evidence of mosaicism also was observed in the red-eyed/CFP^+^ mutant *cd* allele/AgNosCd-1 hemizygous intercross as offspring with apparent wild-type (black) eyes were observed, despite no black-eye phenotypes in the parents. These phenotypes were a result of hidden functional NHEJ alleles that must have been present in the parental germline but not in the somatic cells that give rise to the eye. Importantly, although the red-eyed/CFP^+^ mutant *cd* allele/AgNosCd-1 hemizygous individuals generated as a result of embryonic Cas9 activity were mosaic for multiple resistant alleles, we did not see evidence of true wild-type alleles in these mosaic insects. The reduced bias of the drive alleles in the crosses also supports the conclusion that mosaicism did not likely include any wild-type alleles.

The dynamics of the resistance alleles evaluated here in mixed populations consisting of either gene-drive alleles or wild-type alleles did not give any clear evidence of any fitness cost, benefit, or increased load of the resistance alleles. It was anticipated that a gene-drive allele might carry more of a load compared to a nonfunctional resistance allele due to the potentially negative fitness effect of expression of the gene drive elements, yet it was observed over multiple generations of a mixed drive allele and resistant allele population that both the drive allele and resistance alleles remained near their expected proportions based on Mendelian inheritance.

The creation of the homozygous *cd* resistant Ag*cd*^Δ^*^11^* strain allows us to address questions of any effect the mutations in *cd* alone may have on overall fitness in *An. gambiae* and impact on gene-drive dynamics. The enzyme, HPX6, encoded by the *cd* gene (AGAP003502) is annotated in VectorBase as a heme peroxidase and has a catalytic role in the tryptophan metabolism pathway where it is involved in one of the last steps in the formation of eye pigments (Carballar-Lejarazú *et al.* 2020). Mutations in genes encoding enzymes in the tryptophan metabolic pathway can lead to a number of deleterious pleiotropic effects in insects (Linzen, 1974; Savvateeva-Popova *et al.* 2003; Osanai-Futahashi *et al.* 2016; Zhang *et al.* 2017; Xu *et al.* 2020; Zhuravlev *et al.* 2020). For example, mutations in the gene encoding the kynurenine hydroxylase enzyme (also called kynurenine mono-oxygenase) are orthologs of the *cinnabar* (*cn*) gene and produce a white-eye phenotype in *Aedes aegypti*, *Culex quinquefasciatus* and *An. stephensi* (Cornel *et al.* 1997; Han *et al.* 2003; Gantz *et al.* 2015; Feng *et al.* 2021). Female mosquitoes of these species with spontaneous and induced mutations in their respective *cn* orthologs have impaired survival and reproductive phenotypes that vary in type and severity among the species (Bottino-Rojas *et al.* 2022).

The red color of the eyes in *An. gambiae* homozygous mutant *cd* larval, pupal and newly emerged mosquitoes is an indication of the reduced levels of eye pigments in these insects (Carballar-Lejarazú *et al.* 2020). However, the fact that they have any color at all likely results from the nonenzymatic conversion of the precursor, 3-hydroxykynureine, into an ommochrome, albeit with a titer that is less than that in wild-type mosquitoes. This interpretation is consistent with the observation that the eye color darkens to near-wild in mutant adults within 2 days after eclosion, presumably as the nonenzymatic conversion continues in this stage. A similar phenotype was observed in a mutant line of *Ae. aegypti* (Chen *et al.* 2021). Furthermore, analysis of the accumulation of metabolic products in mosquitoes carrying homozygous gene-drive disruptions of the *cd* locus showed a significantly higher accumulation of xanthurenic acid than controls, presumably resulting from the efficient enzymatic conversion of 3-hydroxykynurenine (Carballar-Lejarazú *et al.* 2020).

Molecularly characterized, nonfunctional *cd* mutations have not been reported in wild-derived *An. gambiae*. However, a spontaneous mutation, *red-eye* (*r*), with a similar phenotype has been described in this species and similar naturally-derived mutations, *crimson* in *Culex*  *pipiens* and *red-eye* (*re*) in *Ae. aegypti* have been described (Bhalla 1968; Beard *et al.* 1995; Rasgon and Scott 2004). Linkage assessments of *crimson* and *re* (3L and sex-linked, respectively) are inconsistent with these being true orthologs of the *Agcd* locus, which was assigned to 2R (VectorBase), although it is likely that synteny for these chromosomes is not conserved among these species. In particular, *re* linked to first chromosome, was shown to be an *Ae. aegypti cd* ortholog using CRISPR gene knock-out tools (Chen *et al.* 2021).

Impacts on fitness from null mutations in the various red-eye phenotype mosquitoes differ among studies. For example, both positive and negative effects have been reported for *Ae. aegypti* while negative effects only were described in *C. pipiens* (Lorimer *et al.* 1976; Wood *et al.* 1977; Rasgon and Scott 2004). While it is assumed that wild-type individuals are likely to out-compete mutant *cd*, drive-resistant individuals in a mixed population over multiple generations, our data are consisted with no negative impact on the reproductive success for mutant *cd* individuals in the laboratory environment where they compete equally with wild-type counterparts. Future semi-field studies will need to be completed in order to determine if *cd* individuals can compete with wild-type individuals outside of the laboratory where factors such as natural sunlight and larval predation can be mimicked and fitness costs re-assessed. However, it is likely that the strength of the drive bias will compensate for any load imposed by the system.

In summary, we have shown that inheritance through maternal lineages can result in the generation of potentially drive-resistant target sites in the gene-drive mosquitoes tested here. However, these observations do not preclude the use of this or a similar system for further development as the basis of a population modification system for controlling mosquito-borne pathogens.

## Data availability

All mosquito strains and plasmids are available upon request. The authors affirm that all data necessary for confirming the conclusions of the article are present within the article, figures, and tables.

Supplemental material is available at *GENETICS* online.

RC-L, TT, TBP, and AAJ designed experiments; RC-L, TT, and TBP conducted experiments; RC-L, TT, TBP, and AAJ analyzed data, and RC-L, TT, TBP, and AAJ prepared the manuscript.

## Supplementary Material

iyac055_Supplementary_Material
