# Supplementary material for: Cas9-mediated maternal effect and derived resistance alleles in a gene-drive strain of the African malaria vector mosquito, Anopheles gambiae
Source: Genetics. 2022 Apr 7;221(2):iyac055. doi: 10.1093/genetics/iyac055 (PMC9157122; doi:10.1093/genetics/iyac055)
Supplement: iyac055_Supplementary_Material [file iyac055_supplementary_material.docx]

**Supporting Information**

**Cas9-mediated maternal-effect and derived resistance alleles in a gene-drive strain of the African malaria vector mosquito, *Anopheles gambiae*, Rebeca Carballar-Lejarazú, Taylor Tushar, Thai Binh Pham and Anthony A. James.**

**Table S1. *Anopheles gambiae* *cardinal* gene (AGAP003502) CDS.**

**Table S2. Progeny from crosses to generate potential drive-resistant *cd* mutant alleles with hemizygous AgNosCd-1 females (Figure 2A).**

**Table S3. Progeny from crosses to generate potential drive-resistant *cd* mutant alleles with homozygous AgNosCd-1 females (Figure 2B).**

**Table S4. Progeny phenotypes and genotypes of red-eye/CFP^+^ progeny from AgNosCd-1 homozygous and hemizygous female outcrosses (Figure 2A and 2B).**

**Table S5. Sanger sequencing results from black-eye/CFP^-^ mosquitoes progeny of hemizygous AgNosCd-1 outcrosses (Figure 2A).**

**Table S6. Progeny phenotypes and genotypes from potential NHEJ resistance-allele/AgNosCd-1 hemizygote intercrosses (Figure 2D).**

**Table S7. Progeny phenotypes and genotypes from potential NHEJ resistance-allele/AgNosCd-1 homozygote or heteroallelic intercrosses (Figure 2E).**

**Table S8. Sequence verification of the red-eye, CFP^-^, *cd* drive-resistant line Ag*cd*^Δ11,14^.**

**Table S9. Phenotypic proportions of progeny of a cross of homozygous resistant line Ag*cd ^Δ11,14^* males to homozygous female AgTP13.**

**Table S10. Phenotypic proportions of progeny of crosses of homozygous resistant line Ag(*cd^Δ11^*) males to female X1-WT.**

**Figure S1. Mosaic eye phenotypes in AgNosCd-1 pupae.**

| **Table S1. *Anopheles gambiae cardinal* gene (AGAP003502) CDS.** |
| --- |
| ATGGTCATGGTTGACGAACGAACGCCACTAACGTCGGACCTTTCCGGTCCGCTTCCGCTGGCGTCGGGACCCTCGGGCACCGCCGTGCATCATCTGAAGTCACACGAAAGTGTTCGGGAGCGCCAGGTCCGAACGTTCCAGTGCTGGATTTGTTCCGCAATACTGGGTGCATTTGCGCTGGCGATCGTGATCAGCATTAGTTACATCATATTCGGCGATGCAACGCAACCACCGCTGGATGGAGCGAACACGACGGCGGCGGATTTTCCTGAGCTCTTAAATCTCATTAGTTTCCCGCTGGTCGATGAGTCGTCACCCGAGTGGAACGGTACGGC**GGTTAGCGACGATGCCAAGG*CGG***CCGCCATAGCGGATGGCGAGAAGGCGCTGGGCGATAAGGAGCTGCTGGAGGAGACGCTCTCGTCGCCCCCGCTTAACTCGCCCTCCTTCCGCCACCAAAAGTCCGTCGGTGCAACGAAAGCGGCCCGGCTGGCGGCAAAGGTCGGCTTCGTGGAGGATCGTGCCACGCAGGCACTGGTGCGCCGGGTGGACATCCGTCGTCGGGGCTCGATCGGGCGCGGCCCACCGATGGATTTGCCGCGTGCGCACCGTCAACCGCGCTGTGACTTTAACGCGCGCTATCGCACGGCCAACGGGACGTGCAACAGTAAGGAGCGACCGTACGAGTACGGCGTCGCGATGATACCGTTCCGGCGCCAGCTCAACCCGGACTATGGCGATGGCATATCGGCACCGCGTGCCTCCGTCGATGGGGCCGAACTGCCCAGCGCCCGGCAGGTGTCGCTCGAAATCCATCGCCCATCGTACCACAACGATCCGAACTTTAGCGTGATGCTGGCGGTGTGGGGCCAGTTTCTCGATCACGACATAACGTCCACCGCGCTGAACCAGGGCGTGGACGGGAAGCCGATCGAGTGCTGCGATCCGGGGCAGCCGCAACATCCCGAGTGCTTTCCCGTGCCGCTCGGTCCGGGCGACCCGTACTACACCCAGTACAACGTGACCTGCATGAACTTTGTGCGCTCGGTGCCGGCCCCGACCGGTCACTTCGGTCCGCGGCAGCAGCTGAACCAGGCGACCGCCTTCATCGACGGCTCGGTCGTGTACGGCTCGGACGACGAGCGTATGGGCGCGCTGCGCACGGGCGCCGGCGGCCAGCTGCGTATGCTGCGCACGCCGGACGGTCGCGATCTGCTGCCCGTGTCGACCGATCCGCTCGACGGGTGCAACGAGCAGGAGATGAATGCGGCCGGCAAGTACTGCTTCGAGTCGGGCGATGCGCGGGCGAACGAGAATCTGCATCTCACCTCGATGCATCTGATCTGGGCCCGGCACCACAACAGCCTTGCCCGCGGGCTGGCCCGCGCAAACCCACACTGGGACGACGAGCGGCTGTTCCAGGAGGCGCGCCGCATCCTGGCCGCCCAGATGCAGCACATCACGTACGCCGAGTTTGTGCCGGTGATCGTCGGCAACGAGACGGCCGGCCGGATGGGCCTGCTGCCCGTATCGGCCGGTGGCGAGCCGGCCGGCGACACGTACAACGCCACGGTGGACGCGTCGATCGCGAACGTGTTTGCCGGGGCCGCGTTCCGCTTCGCCCACACGCTGCTGCCCGGGCTGATGAAGCAAACGCGCAATCCGGCCGCGTCCGCCTCGGGCATCGAGCTGCACCGGATGCTGTTCAACCCGTACTCGCTGTACGCGCGCGACGGGCTGGACAATGCGCTGGGCGGTGCGATCGGTACGGCGCTGGCCAAGTACGACCAGTACTTCTCCACCGAGCTGACCGAGCGGCTGTTCGAGAAGGCGGACGAGCATCTGCTGCACGGCCAGCCGTGCGGGTTGGATCTGGTGTCGCTCAACATACAGCGCGGCCGGGACCATGGGCTGCCGGCGTACCCGCGCTGGCGCAAGCACTGCCACCTGACGCCGGCCGACAGCTGGGAGGAGCTGGAGCGCATCGTCGATCCGGAATCGTACCGGCAGATGAGGCGCATCTACCGCGAGCCGGCCAACGTGGACGTTTACTCCGGCGCGCTCAGTGAAGCGCCGGTGCGCGATGGGATCGTCGGGCCGCTGCTGACGTGCCTGATTGGCGATCAGTTTTTGCGGCTAAAGCAGGGCGACTCGTTCTGGTACGAGAGACGGCGGGGACCGCAACGGTTCACCGAAGCACAATTGCAGCAAATTTACAACACCAAACTGTCGAGCATTATTTGTCGCAATTCGGACCACATTGAGCAGTCGCCCGTGTATCTGATGAAGCGTACCGATTCCCGCACGAACCCGGAAACGGACTGCAAGCAGCTGGACACGTTTGATTTCGAGCCGTTCCGAGAGGACGCCGAACAGCCGCAGCGCAACCGGGCGGCCAAAATTGCGACCGACCGCATGAAGGTGCTGGTGTTCGAACCGAAGGCAACCGGAACCACCACCGAGCACGCGGTCGGGGCAGAAATGCAGGACGTGGAGGAGGGAGAGCGCCAAGCGGCCAGCACGACGACGGTGGCAACGACAACGGATGCGACGACGACGACGACGACAACGACGACCACGATGAAGGAGGCTGCAGGAGCATGA |
| The duplicated sequences identified in black-eye/CFP^-^ individuals (**Table S7**) are highlighted in green and underlined.  gRNA target, bold and yellow highlighted; PAM: underlined and italics. |

| **Table S2. Progeny from crosses to generate potential drive-resistant *cd* mutant alleles with hemizygous AgNosCd-1 females (Figure 2A).** | | | | | | | |
| --- | --- | --- | --- | --- | --- | --- | --- |
| **Phenotype** | **Rep. 1** |  | **Rep. 2** |  | **Rep. 3** |  | **Total** |
| **WT eye/CFP^+^** | 515 |  | 171 |  | 64 |  | **750** |
| **WT eye/CFP^-^** | 12 |  |  |  | 1 |  | **13** |
| **Mosaic (Tear) eye/CFP^+^** | 127 |  | 55 |  | 11 |  | **193** |
| **Red eye (*cd*) /CFP^+^** | 9 |  | 1 |  | 0 |  | **10** |
| **Total** | **663** |  | **227** |  | **76** |  | **966** |
| Replicates (Reps) are 50 hemizygous AgNosCd-1 females crossed with 50 wild-type males in triplicate. WT, wild-type (black) eye color; CFP^+^, cyan fluorescent protein positive; CFP^-^, cyan fluorescent protein negative; *cd*, *cardinal* red-eye. | | | | | | | |

| **Table S3. Progeny from crosses to generate potential drive-resistant *cd* mutant alleles with homozygous AgNosCd-1 females (Figure 2B).** | | | | | | | |
| --- | --- | --- | --- | --- | --- | --- | --- |
| **Phenotype** | **Rep. 1** |  | **Rep. 2** |  | **Rep. 3** |  | **Total** |
| **WT eye/CFP^+^** | 144 |  | 113 |  | 23 |  | **280** |
| **Mosaic (Tear) eye/CFP^+^** | 191 |  | 218 |  | 32 |  | **441** |
| **Red eye (*cd*) /CFP^+^** | 19 |  | 26 |  | 2 |  | **47** |
| **Total** | **354** |  | **357** |  | **57** |  | **768** |
| Replicates (Reps) are 50 homozygous AgNosCd-1 females crossed with 50 wild-type males in triplicate. WT, wild-type (black) eye color; CFP^+^, cyan fluorescent protein positive; CFP^-^, cyan fluorescent protein negative; *cd*, *cardinal* red-eye. | | | | | | | |

| **Table S4. Progeny phenotypes and genotypes of red-eye/CFP^+^ progeny from AgNosCd-1 homozygous and hemizygous female outcrosses (Figure 2A and 2B).** | | | |
| --- | --- | --- | --- |
|  |  | **Genotypes (DNA and amino acid sequences)** |  |
| **Parent pheno/geno** | **Prog pheno** | **WT acggcggttagcgacgatgccaaggcggccgccatagcggatggcgagaaggcgctgggcgataaggagctgctggaggagacgctctcgtcgcccccgcttaactcgccctccttccgccaccaaaag**  **T A  V  S  D  D  A  K  A  A  A  I  A  D  G  E  K  A  L  G  D K  E  L  L  E  E  T  L  S  S  P  P  L  N  S  P  S  F  R  H Q  K** | **F** |
| **black-eye/ CFP^-^ males**  **X**  **red-eye/ CFP^+^ females**  **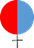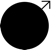**  **W/W D/D** | **red-eye/ CFP^+^**  **males and females**  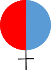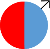 | **1. acggcggttagcgacgatgcc-ggcggtcagcgacgaggcggccgccatagcggatggcgagaaggcgctgggcgataaggagctgctggaggagacgctctcgtcgcccccgcttaactcgccctccttccgccaccaaaag**  **T A V S D D A G G Q R R G G R H S G W R E G A G R * G A G G D A L V A P A * L A L L P P P K**  **2.a. acggcggttagcgacgatgaca-----------gccatagcggatggcgagaaggcgctgggcgataaggagctgctggaggagacgctctcgtcgcccccgcttaactcgccctccttccgccaccaaaag**  **T A  V  S  D  D  D S H S G W R E G A G R * G A A G G D A L V A P A * L A L L P P P K**  **2.b. acggcggttagcgaccgt--------------cgccatagcggatggcgagaaggcgctgggcgataaggagctgctggaggagacgctctcgtcgcccccgcttaactcgccctccttccgccaccaaaag**  **T A  V  S  D  R  R H S G W R E G A G R * G A A G G D A L V A P A * L A L L P P P K**  **3. acggcggttagcgac--------------cgccatagcggatggcgagaaggcgctgggcgataaggagctgctggaggagacgctctcgtcgcccccgcttaactcgccctccttccgccaccaaaag**  **T A  V  S  D R H S G W R E G A G R * G A A G G D A L V A P A * L A L L P P P K**  **4.a. acggcggttagcgacgatgc--aggcggccgccatagcggatggcgagaaggcgctgggcgataaggagctgctggaggagacgctctcgtcgcccccgcttaactcgccctccttccgccaccaaaag**  **T A  V  S  D  D  A   G G R H S G W R E G A G R * G A A G G D A L V A P A * L A L L P P P K**  **4.b. acg-----------------------ctatcgtcgcaccggtggtaggccgccatagcggatggcgagaaggcgctgggcgataaggagctgctggaggagacgctctcgtcgcccccgcttaactcgccctccttccgccaccaaaag**  **T L S S H R W * A A I A D G E K A L G D K E L L E E T L S S P P L N S P S F R H Q K**  **5. acggcggttagcgacgatgcggat---ggcggccgccatagcggatggcgagaaggcgctgggcgataaggagctgctggaggagacgctctcgtcgcccccgcttaactcgccctccttccgccaccaaaag**  **T A  V  S  D  D  A D G G R H S G W R E G A G R * G A A G G D A L V A P A * L A L L P P P K**  **6. acggcggttagcgacgatgccatcgtcc-------------------------------------------------------------------------------taactcgccctccttccgccaccaaaag**  **T A  V  S  D  D  A I V L T R P P S A T K S**  **7. cccgagtggaa---------------------------ggcggccgccatagcggatggcgagaaggcgctgggcgataaggagctgctggaggagacgctctcgtcgcccccgcttaactcgccctccttccgccaccaaaag**  **P E W K A  A  A  I  A  D  G  E  K  A  L  G  D K  E  L  L  E  E  T  L  S  S  P  P  L  N  S  P  S  F  R  H Q  K**  **8.a. acggcggttagcgacgatg-------------------------gcgagaaggcgctgggcgataaggagctgctggaggagacgctctcgtcgcccccgcttaactcgccctccttccgccaccaaaag**  **T A  V  S  D  D    G E  K  A  L  G  D K  E  L  L  E  E  T  L  S  S  P  P  L  N  S  P  S  F  R  H Q  K**  **8.b. acggcggttagcgacgatgccaggagggggacgaggcggccgccatagcggatggcgagaaggcgctgggcgataaggagctgctggaggagacgctctcgtcgcccccgcttaactcgccctccttccgccaccaaaag**  **T A  V  S  D  D  A R R G T R R P P  * R M A R R R W A I R S C W R R R S R R P R L T R P P S A T K S**  **9.a. acggcggttagcgacgatgcca----------------ggatggcgagaaggcgctgggcgataaggagctgctggaggagacgctctcgtcgcccccgcttaactcgccctccttccgccaccaaaag**  **T A  V  S  D  D  A R M A R R R W A I R S C W R R R S R R P R L T R P P S A T K S**  **9.b. acggcggttagcgacg------aggcggccgccatagcggatggcgagaaggcgctgggcgataaggagctgctggaggagacgctctcgtcgcccccgcttaactcgccctccttccgccaccaaaag**  **T A  V  S  D  E   A  A  A  I  A  D  G  E  K  A  L  G  D K  E  L  L  E  E  T  L  S  S  P  P  L  N  S  P  S  F  R  H Q  K**  **10. acggcggttagcgacgatg-----gcggccgccatagcggatggcgagaaggcgctgggcgataaggagctgctggaggagacgctctcgtcgcccccgcttaactcgccctccttccgccaccaaaag**  **T A  V  S  D  D  G G R H S G W R E G A G R * G A A G G D A L V A P A * L A L L P P P K**  **11. acggcggttagcgacgata------gcggccgccatagcggatggcgagaaggcgctgggcgataaggagctgctggaggagacgctctcgtcgcccccgcttaactcgccctccttccgccaccaaaag**  **T A  V  S  D  D   S G R H S G W R E G A G R * G A A G G D A L V A P A * L A L L P P P K**  **12. acggcggttagcg--------------------------gatggcgagaaggcgctgggcgataaggagctgctggaggagacgctctcgtcgcccccgcttaactcgccctccttccgccaccaaaag**  **T A  V  S  G   W R E G A G R * G A A G G D A L V A P A * L A L L P P P K**  **13. acggcggttagcgacgat---aatccgctaaggcggccgccatagcggatggcgagaaggcgctgggcgataaggagctgctggaggagacgctctcgtcgcccccgcttaactcgccctccttccgccaccaaaag**  **T A V S D D N P L R R P P * R M A R R R W A I R S C W R R R S R R P R L T R P P S A T K S**  **14.a. acggcggttagcgacgat---------gccgccatagcggatggcgagaaggcgctgggcgataaggagctgctggaggagacgctctcgtcgcccccgcttaactcgccctccttccgccaccaaaag**  **T A  V  S  D  D    A  A  I  A  D  G  E  K  A  L  G  D K  E  L  L  E  E  T  L  S  S  P  P  L  N  S  P  S  F  R  H Q  K**  **14.b. ac-----------------------cggccgccatagcggatggcgagaaggcgctgggcgataaggagctgctggaggagacgctctcgtcgcccccgcttaactcgccctccttccgccaccaaaag**  **T G R H S G W R E G A G R * G A A G G D A L V A P A * L A L L P P P K**  **15. acggcggttagcgacgatgcc-----ttagggccgccatagcggatggcgagaaggcgctgggcgataaggagctgctggaggagacgctctcgtcgcccccgcttaactcgccctccttccgccaccaaaag**  **T A  V  S  D  D  A   L G P P * R M A R R R W A I R S C W R R R S R R P R L T R P P S A T K S**  **16. acggcggttagcgacgatgcc-tcatgaggcggccgccatagcggatggcgagaaggcgctgggcgataaggagctgctggaggagacgctctcgtcgcccccgcttaactcgccctccttccgccaccaaaag**  **T A  V  S  D  D  A   S * G G R H S G W R E G A G R * G A A G G D A L V A P A * L A L L P P P K**  **17.a. acggcggttagcgacgatgccataggcggcagaggcggccgccatagcggatggcgagaaggcgctgggcgataaggagctgctggaggagacgctctcgtcgcccccgcttaactcgccctccttccgccaccaaaag**  **T A  V  S  D  D  A  I G G R G G R H S G W R E G A G R * G A A G G D A L V A P A * L A L L P P P K**  **17.b. acggcggttagcgacgatgccgttag-----ggccgccatagcggatggcgagaaggcgctgggcgataaggagctgctggaggagacgctctcgtcgcccccgcttaactcgccctccttccgccaccaaaag**  **T A  V  S  D  D  A  V R  A  A  I  A  D  G  E  K  A  L  G  D K  E  L  L  E  E  T  L  S  S  P  P  L  N  S  P  S  F  R  H Q  K**  **18. acggcggttagcgacgatgcca-ggcggccgccatagcggatggcgagaaggcgctgggcgataaggagctgctggaggagacgctctcgtcgcccccgcttaactcgccctccttccgccaccaaaag**  **T A  V  S  D  D  A  R R P P * R M A R R R W A I R S C W R R R S R R P R L T R P P S A T K** | **O**  **O**  **O**  **O**  **O**  **O**  **O**  **O**  **I**  **O**  **O**  **O**  **I**  **O**  **O**  **I**  **I**  **I**  **O**  **O**  **O**  **O**  **I**  **O** |
| **black-eye/ CFP^-^ males**  **X**  **black-eye/ CFP^+^ females**  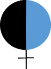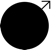  **W/W W/D** | **red-eye/ CFP^+^**  **males and females**  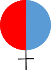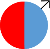 | **19.a. acggcggttagcgacgatg--------gccgccatagcggatggcgagaaggcgctgggcgataaggagctgctggaggagacgctctcgtcgcccccgcttaactcgccctccttccgccaccaaaag**  **T A  V S D D   G R H S G W R E G A G R * G A A G G D A L V A P A * L A L L P P P K**  **19.b. acgg---------------------cggccgccatagcggatggcgagaaggcgctgggcgataaggagctgctggaggagacgctctcgtcgcccccgcttaactcgccctccttccgccaccaaaag**  **T A   A A I  A  D  G  E  K  A  L  G  D K  E  L  L  E  E  T  L  S  S  P  P  L  N  S  P  S  F  R  H Q  K**  **20. acggcggttagcgacgatg-----gcggccgccatagcggatggcgagaaggcgctgggcgataaggagctgctggaggagacgctctcgtcgcccccgcttaactcgccctccttccgccaccaaaag**  **T A  V  S  D  D  G G R H S G W R E G A G R * G A A G G D A L V A P A * L A L L P P P K**  **21. acggcggttagcgacgat----aggcggccgccatagcggatggcgagaaggcgctgggcgataaggagctgctggaggagacgctctcgtcgcccccgcttaactcgccctccttccgccaccaaaag**  **T A  V  S  D  D   R R P P * R M A R R R W A I R S C W R R R S R R P R L T R P P S A T K S**  **22.a. acggcggttagcgacgatgcg--aggcggccgccatagcggatggcgagaaggcgctgggcgataaggagctgctggaggagacgctctcgtcgcccccgcttaactcgccctccttccgccaccaaaag**  **T A  V  S  D  D  A   R R P P * R M A R R R W A I R S C W R R R S R R P R L T R P P S A T K S**  **22.b. acggcggttagcgacgaagcg-----aggcggccgccatagcggatggcgagaaggcgctgggcgataaggagctgctggaggagacgctctcgtcgcccccgcttaactcgccctccttccgccaccaaaag**  **T A  V  S  D  E A   R R P P * R M A R R R W A I R S C W R R R S R R P R L T R P P S A T K S** | **O**  **I**  **O**  **O**  **O**  **O** |
| **Sequences include the region of DNA containing the AgNosCd-1 Cas9/gRNA target site and PAM sequence, wild-type (WT) *An. gambiae* G3 reference sequence is shown for comparison. Frame indicate in-frame or O-of-frame mutation. Corresponding amino acids are shown below each codon in the sequence. Numbers (1-22) represent individual mosquito; letters (a, b) represent individual alleles. Blue: gRNA sequence; brown: PAM; red: deletion; purple: insertion; *: stop codon, W = wild-type allele, D = drive allele, pheno: phenotype, geno: genotype, Prog: progeny, F: frame, O: Out, I: in. Eye phenotypes described in Figure 2.** | | | |

| **Table S5. Sanger sequencing results from black-eye/CFP^-^ mosquitoes progeny of hemizygous AgNosCd-1 outcrosses (Figure 2A).** |
| --- |
| .NNNTATTGTTGTNNCAGATGAGTCGTCACCCGAGTGGAACGGTACGGC**GGTTAGCGACGATGCCAAGG*CGG***CCGCCATAGCGGATGGCGAGAAGGCGCTGGGCGATAAGGAGCTGCTGGAGGAGACGCTCTCGTCGCCCCCGCTTAACTCGCCCTCCTTCCGCCACCAAAAGTCCGTCGGTGCAACGAAAGCGGCCCGGCTGGCGGCAAAGGTCGGCTTCGTGGAGGATCGTGCCACGCAGGCACTGGTGCGCCGGTTGGACATCCGTCGTCGGGGCTCGATCGGGCGCGGCCCACCGATGGATTTGCCGCGTGCGCACCGTCAACCGCGCTGCGACTTCAACGCGCGCTATCGCACNCNNNNNNNNNN |
| ...NNTATTGTTNCTNNNATGAGTCGTCACCCGAGTGGAACGGTACGGC**GGTTAGCGACGATGCCAAGG*CGG***CCGCCATAGCGGATGGCGAGAAGGCGCTGGGCGATAAGGAGCTGCTGGAGGAGACGCTCTCGTCGCCCCCGCTTAACTCGCCCTCCTTCCGCCACCAAAAGTCCGTCGGTGCAACGAAAGCGGCCCGGCTGGCGGCAAAGGTCGGCTTCGTGGAGGATCGTGCCACGCAGGCACTGGTGCGCCGGTTGGACATCCGTCGTCGGGGCTCGATCGGGCGCGGCCCACCGATGGATTTGCCGCGTGCGCACCGTCAACCGCGCTGCGACTTCAACGCGCGCTATCGCACNNCANNNNNNN |
| .NNNTTATTGTTNNNNCANTGAGTCGTCACCCGAGTGGAACGGTACGGC**GGTTAGCGACGATGCCAAGG*CGG***CCGCCATAGCGGATGGCGAGAAGGCGCTGGGCGATAAGGAGCTGCTGGAGGAGACGCTCTCGTCGCCCCCGCTTAACTCGCCCTCCTTCCGCCACCAAAAGTCCGTCGGTGCAACGAAAGCGGCCCGGCTGGCGGCAAAGGTCGGCTTCGTGGAGGATCGTGCCACGCAGGCACTGGTGCGCCGGTTGGACATCCGTCGTCGGGGCTCGATCGGGCGCGGCCCACCGATGGATTTGCCGCGTGCGCACCGTCAACCGCGCTGCGACTTCAACGCGCGCTATCGCACNNCANNNNNNN |
| ...NCTATTGTTGNTNNNATGAGTCGTCACCCGAGTGGAACGGTACGGC**GGTTAGCGACGATGCCAAGG*CGG***CCGCCATAGCGGATGGCGAGAAGGCGCTGGGCGATAAGGAGCTGCTGGAGGAGACGCTCTCGTCGCCCCCGCTTAACTCGCCCTCCTTCCGCCACCAAAAGTCCGTCGGTGCAACGAAAGCGGCCCGGCTGGCGGCAAAGGTCGGCTTCGTGGAGGATCGTGCCACGCAGGCACTGGTGCGCCGGTTGGACATCCGTCGTCGGGGCTCGATCGGGCGCGGCCCACCGATGGATTTGCCGCGTGCGCACCGTCAACCGCGCTGCGACTTCAACGCGCGCTATCGCACNNCNNNNNNNN |
| ..NGTTATTGTTGNNNNNATGAGTCGTCACCCGAGTGGAACGGTACGGC**GGTTAGCGACGATGCCAAGG*CGG***CCGCCATAGCGGATGGCGAGAAGGCGCTGGGCGATAAGGAGCTGCTGGAGGAGACGCTCTCGTCGCCCCCGCTTAACTCGCCCTCCTTCCGCCACCAAAAGTCCGTCGGTGCAACGAAAGCGGCCCGGCTGGCGGCAAAGGTCGGCTTCGTGGAGGATCGTGCCACGCAGGCACTGGTGCGCCGGTTGGACATCCGTCGTCGGGGCTCGATCGGGCGCGGCCCACCGATGGATTTGCCGCGTGCGCACCGTCAACCGCGCTGCGACTTCAACGCGCGCTATCGCACNNCANNNNNNN |
| ..NNTTATTGTTNANNGNATGAGTCGTCACCCGAGTGGAACGGTACGGC**GGTTAGCGACGATGCCAAGG*CGG***CCGCCATAGCGGATGGCGAGAAGGCGCTGGGCGATAAGGAGCTGCTGGAGGAGACGCTCTCGTCGCCCCCGCTTAACTCGCCCTCCTTCCGCCACCAAAAGTCCGTCGGTGCAACGAAAGCGGCCCGGCTGGCGGCAAAGGTCGGCTTCGTGGAGGATCGTGCCACGCAGGCACTGGTGCGCCGGTTGGACATCCGTCGTCGGGGCTCGATCGGGCGCGGCCCACCGATGGATTTGCCGCGTGCGCACCGTCAACCGCGCTGCGACNNAACGCGCGCTATCGCACNNCANNNNNNNN |
| ………..NATTGTTGCNCAGATGAGTCGTCACCCGAGTGGAACGGTACGGC**GGTTAGCGACGATGCCAAGG*CGG***CCGCCATAGCGGATGGCGAGAAGGCGCTGGGCGATAAGGAGCTGCTGGAGGAGACGCTCTCGTCGCCCCCGCTTAACTCGCCCTCCTTCCGCCACCAAAAGTCCGTCGGTGCAACGAAAGCGGCCCGGCTGGCGGCAAAGGTCGGCTTCGTGGAGGATCGTGCCACGCAGGCACTGGTGCGCCGGTTGGACATCCGTCGTCGGGGCTCGATCGGGCGCGGCCCACCGATGGATTTGCCGCGTGCGCACCGTCAACCGCGCTGCGANNNAACGCGCGCTATCGCACNNCNNNNNNNNN |
| .NCTTATTGTTNNNAACANTGAGTCGTCACCCGAGTGGAACGGTACGGC**GGTTAGCGACGATGCCAAGG*CGG***CCGCCATAGCGGATGGCGAGAAGGCGCTGGGCGATAAGGAGCTGCTGGAGGAGACGCTCTCGTCGCCCCCGCTTAACTCGCCCTCCTTCCGCCACCAAAAGTCCGTCGGTGCAACGAAAGCGGCCCGGCTGGCGGCAAAGGTCGGCTTCGTGGAGGATCGTGCCACGCAGGCACTGGTGCGCCGGTTGGACATCCGTCGTCGGGGCTCGATCGGGCGCGGCCCACCGATGGATTTGCCGCGTGCGCACCGTCAACCGCGCTGCGACTCAACGCGCGCTATCGCACNNCNNNNNNNN |
| ...NNTATTGTNTNCNNNATGAGTCGTCACCCGAGTGGAACGGTACGGC**GGTTAGCGACGATGCCAAGG*CGG***CCGCCATAGCGGATGGCGAGAAGGCGCTGGGCGATAAGGAGCTGCTGGAGGAGACGCTCTCGTCGCCCCCGCTTAACTCGCCCTCCTTCCGCCACCAAAAGTCCGTCGGTGCAACGAAAGCGGCCCGGCTGGCGGCAAAGGTCGGCTTCGTGGAGGATCGTGCCACGCAGGCACTGGTGCGCCGGTTGGACATCCGTCGTCGGGGCTCGATCGGGCGCGGCCCACCGATGGATTTGCCGCGTGCGCACCGTCAACCGCGCTGCGACTTCAACGCGCGCTATCGCACNNCNNNNNNNN |
| ..NNTATTGTTGNNNCAGATGAGTCGTCACCCGAGTGGAACGGTACGGC**GGTTAGCGACGATGCCAAGG*CGG***CCGCCATAGCGGATGGCGAGAAGGCGCTGGGCGATAAGGAGCTGCTGGAGGAGACGCTCTCGTCGCCCCCGCTTAACTCGCCCTCCTTCCGCCACCAAAAGTCCGTCGGTGCAACGAAAGCGGCCCGGCTGGCGGCAAAGGTCGGCTTCGTGGAGGATCGTGCCACGCAGGCACTGGTGCGCCGGTTGGACATCCGTCGTCGGGGCTCGATCGGGCGCGGCCCACCGATGGATTTGCCGCGTGCGCACCGTCAACCGCGCTGCGACTTCAACGCGCGCTNTCGCACNNCNNNNNNNN |
| NGGTTATTGTTGNANCAGATGAGTCGTCACCCGAGTGGAACGGTACGGC**GGTTAGCGACGATGCCAAGG*CGG***CCGCCATAGCGGATGGCGAGAAGGCGCTGGGCGATAAGGAGCTGCTGGAGGAGACGCTCTCGTCGCCCCCGCTTAACTCGCCCTCCTTCCGCCACCAAAAGTCCGTCGGTGCAACGAAAGCGGCCCGGCTGGCGGCAAAGGTCGGCTTCGTGGAGGATCGTGCCACCCAGGCACTGGTGCGCCGGCTGGACATCCGTCGTCGGGGCTCGATCGGGCGCGGCCCACCGATGGATTTGCCGCGTGCGCACCGTCAACCGCGCTGCGACTTAACGCGCGCTATCGCACNNCNNNNNNNN |
| ..GNTTATTGTTNNNNNNATGAGTCGTCACCCGAGTGGAACGGTACGGC**GGTTAGCGACGATGCCAAGG*CGG***CCGCCATAGCGGATGGCGAGAAGGCGCTGGGCGATAAGGAGCTGCTGGAGGAGACGCTCTCGTCGCCCCCGCTTAACTCGCCCTCCTTCCGCCACCAAAAGTCCGTCGGTGCAACGAAAGCGGCCCGGCTGGCGGCAAAGGTCGGCTTCGTGGAGGATCGTGCCACGCAGGCACTGGTGCGCCGGTTGGACATCCGTCGTCGGGGCTCGATCGGGCGCGGCCCACCGATGGATTTGCCGCGTGCGCACCGTCAACCGCGCTGCGACTTCAACGCGCGCTATCGCACNCCNNNNNNNN |
| .NATTATTGTTGNTNGNNATGAGTCGTCACCCGAGTGGAACGGTACGGC**GGTTAGCGACGATGCCAAGG*CGG***CCGCCATAGCGGATGGCGAGAAGGCGCTGGGCGATAAGGAGCTGCTGGAGGAGACGCTCTCGTCGCCCCCGCTTAACTCGCCCTCCTTCCGCCACCAAAAGTCCGTCGGTGCAACGAAAGCGGCCCGGCTGGCGGCAAAGGTCGGCTTCGTGGAGGATCGTGCCACGCAGGCACTGGTGCGCCGGTTGGACATCCGTCGTCGGGGCTCGATCGGGCGCGGCCCACCGATGGATTTGCCGCGTGCGCACCGTCAACCGCGCTGCGANNNAACGCGCGCTATCGCACNCCNNNNNNNN |
| All 13 black-eye/CFP^-^ progeny of hemizygous AgNosCd-1 outcrossed to WT males (**Figure 2A**) showed conserved gRNA targets highlighted in yellow and in bold letters. The PAM sequence are underlined and in italics). All sequences are from single mosquito genomic DNA extractions. |

| **Table S6. Progeny phenotypes and genotypes from potential NHEJ resistance-allele/AgNosCd-1 hemizygote intercrosses (Figure 2D).** | | | |
| --- | --- | --- | --- |
|  |  | **Genotypes (DNA and amino acid sequences)** |  |
| **Parent pheno/geno** | **Prog pheno** | **WT acggcggttagcgacgatgccaaggcggccgccatagcggatggcgagaaggcgctgggcgataaggagctgctggaggagacgctctcgtcgcccccgcttaactcgccctccttccgccaccaaaag**  **T A  V  S  D  D  A  K  A  A  A  I  A  D  G  E  K  A  L  G  D K  E  L  L  E  E  T  L  S  S  P  P  L  N  S  P  S  F  R  H Q  K** | **F** |
| **red-eye/ CFP^+^**  **males**  **X**  **red-eye/ CFP^+^**  **females**  **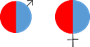**  **D/D, R/D or r/D** | **red-eye/CFP^+^**  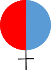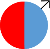 | 1. **acggcggttagcgacgatgccagttagcgaggcggccgccatagcggatggcgagaaggcgctgggcgataaggagctgctggaggagacgctctcgtcgcccccgcttaactcgccctccttccgccaccaaaag**   **T A  V  S  D  D  A  S * R G G R H S G W R E G A G R * G A A G G D A L V A P A * L A L L P P P K**  **2. acggcggttagcgacg-----------gccgccatagcggatggcgagaaggcgctgggcgataaggagctgctggaggagacgctctcgtcgcccccgcttaactcgccctccttccgccaccaaaag**  **T A  V  S  D  G R H S G W R E G A G R * G A A G G D A L V A P A * L A L L P P P K**  **3. acggcggttagcgacgatgcc-ggcggtcagcgacgaggcggccgccatagcggatggcgagaaggcgctgggcgataaggagctgctggaggagacgctctcgtcgcccccgcttaactcgccctccttccgccaccaaaag**  **T A V S D D A G G Q R R G G R H S G W R E G A G R * G A A G G D A L V A P A * L A L L P P P K**  **4. acggcggttagcgacgatgccataggcggcagaggcggccgccatagcggatggcgagaaggcgctgggcgataaggagctgctggaggagacgctctcgtcgcccccgcttaactcgccctccttccgccaccaaaag**  **T A  V  S  D  D  A  I G G R G G R H S G W R E G A G R * G A A G G D A L V A P A * L A L L P P P K**  **5-10. homozygous for drive allele** | **O**  **O**  **O**  **O** |
|  | **red-eye/CFP^-^**  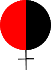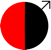 | **1-2.a. acggcggttagcgacgatgccataggcggcagaggcggccgccatagcggatggcgagaaggcgctgggcgataaggagctgctggaggagacgctctcgtcgcccccgcttaactcgccctccttccgccaccaaaag**  **T A  V  S  D  D  A  I G G R G G R H S G W R E G A G R * G A A G G D A L V A P A * L A L L P P P K**  **1-2.b. acggcggttagcgacgactgtactgggtgtagtacgggtcgcccggaccgagcggcacgggaaagcactcgggatgttgcggctgccccggatcgcagcactcgatcggcttcccgtccacgccctccttcgagc-----gaggcggccgcc**  **T A  V  S  D  D C T G C S T G R P D R A A R E S T R D V A A A P D R S T R S A S R P R P P S S E A A A**  **3-4.a. acggcggttagcgacgatgccatcgtcc-------------------------------------------------------------------------------taactcgccctccttccgccaccaaaag**  **T A  V  S  D  D  A I V L T R P P S A T K S**  **3-4.b. acggcggttagcgacgatgccagttagcgaggcggccgccatagcggatggcgagaaggcgctgggcgataaggagctgctggaggagacgctctcgtcgcccccgcttaactcgccctccttccgccaccaaaag**  **T A  V  S  D  D  A  S * R G G R H S G W R E G A G R * G A A G G D A L V A P A * L A L L P P P K**  **5-7.a. acggcggttagcgacgatgccataggcggcagaggcggccgccatagcggatggcgagaaggcgctgggcgataaggagctgctggaggagacgctctcgtcgcccccgcttaactcgccctccttccgccaccaaaag**  **T A  V  S  D  D  A  I G G R G G R H S G W R E G A G R * G A A G G D A L V A P A * L A L L P P P K**  **5-7.b. acggcggttagcgacgatgcc-tcatgaggcggccgccatagcggatggcgagaaggcgctgggcgataaggagctgctggaggagacgctctcgtcgcccccgcttaactcgccctccttccgccaccaaaag**  **T A  V  S  D  D  A   S * G G R H S G W R E G A G R * G A A G G D A L V A P A * L A L L P P P K**  **8.a. acggcggttagcgacgat---------gccgccatagcggatggcgagaaggcgctgggcgataaggagctgctggaggagacgctctcgtcgcccccgcttaactcgccctccttccgccaccaaaag**  **T A  V  S  D  D     A  A  I  A  D  G  E  K  A  L  G  D K  E  L  L  E  E  T  L  S  S  P  P  L  N  S  P  S  F  R  H Q  K**  **8.b. acggcggttagcgacg-----------gccgccatagcggatggcgagaaggcgctgggcgataaggagctgctggaggagacgctctcgtcgcccccgcttaactcgccctccttccgccaccaaaag**  **T A  V  S  D  G R H S G W R E G A G R * G A A G G D A L V A P A * L A L L P P P K**  **9-10.a.acggcggttagcgac------------------------gatggcgagaaggcgctgggcgataaggagctgctggaggagacgctctcgtcgcccccgcttaactcgccctccttccgccaccaaaag**  **T A  V  S  D     D  G  E  K  A  L  G  D K  E  L  L  E  E  T  L  S  S  P  P  L  N  S  P  S  F  R  H Q  K**  **9-10.b.acggcggttagcgacgatgcca-ggcggccgccatagcggatggcgagaaggcgctgggcgataaggagctgctggaggagacgctctcgtcgcccccgcttaactcgccctccttccgccaccaaaag**  **T A  V  S  D  D  A  R R P P * R M A R R R W A I R S C W R R R S R R P R L T R P P S A T K** | **O**  **I**  **O**  **O**  **O**  **O**  **I**  **O**  **I**  **O** |
|  | **black-eye/ CFP^-^**  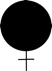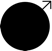 | **1-4.a. acggcggttagcgacgatgcca--tagcggccgccatagcggatggcgagaaggcgctgggcgataaggagctgctggaggagacgctctcgtcgcccccgcttaactcgccctccttccgccaccaaaag**  **T A  V  S  D  D  A  I  A  A  A  I  A  D  G  E  K  A  L  G  D K  E  L  L  E  E  T  L  S  S  P  P  L  N  S  P  S  F  R  H Q  K**  **1-4.b. acggcggttagcgacg-----------gccgccatagcggatggcgagaaggcgctgggcgataaggagctgctggaggagacgctctcgtcgcccccgcttaactcgccctccttccgccaccaaaag**  **T A  V  S  D  G R H S G W R E G A G R * G A A G G D A L V A P A * L A L L P P P K**  **5.a. acggcggttagcgacg------aggcggccgccatagcggatggcgagaaggcgctgggcgataaggagctgctggaggagacgctctcgtcgcccccgcttaactcgccctccttccgccaccaaaag**  **T A  V  S  D  E    A  A  A  I  A  D  G  E  K  A  L  G  D K  E  L  L  E  E  T  L  S  S  P  P  L  N  S  P  S  F  R  H Q  K**  **5.b. acggcggttagcgacgatgc----cggcggtcagcgacgatgcggccgccatagcggatggcgagaaggcgctgggcgataaggagctgctggaggagacgctctcgtcgcccccgcttaactcgccctccttccgccaccaaaag**  **T A  V  S  D  D  A   G  G  Q  R  R  C  G  R  H  S  G  W  R  E  G  A  G  R  * G  A  A  G G D A L V A P A * L A L L P P P K**  **6-7.a. acggcggttagcgacg------aggcggccgccatagcggatggcgagaaggcgctgggcgataaggagctgctggaggagacgctctcgtcgcccccgcttaactcgccctccttccgccaccaaaag**  **T A  V  S  D  E    A  A  A  I  A  D  G  E  K  A  L  G  D K  E  L  L  E  E  T  L  S  S  P  P  L  N  S  P  S  F  R  H Q  K**  **6-7.b. acggcggttagcgacgatgcca-ggcggccgccatagcggatggcgagaaggcgctgggcgataaggagctgctggaggagacgctctcgtcgcccccgcttaactcgccctccttccgccaccaaaag**  **T A  V  S  D  D  A  R R P P * R M A R R R W A I R S C W R R R S R R P R L T R P P S A T K**  **8.a. acggcggttagcgacgat---------gccgccatagcggatggcgagaaggcgctgggcgataaggagctgctggaggagacgctctcgtcgcccccgcttaactcgccctccttccgccaccaaaag**  **T A  V  S  D  D     A  A  I  A  D  G  E  K  A  L  G  D K  E  L  L  E  E  T  L  S  S  P  P  L  N  S  P  S  F  R  H Q  K**  **8.b. acggcggttagcga-----------cggccgccatagcggatggcgagaaggcgctgggcgataaggagctgctggaggagacgctctcgtcgcccccgcttaactcgccctccttccgccaccaaaag**  **T A  V  S  D   G R H S G W R E G A G R * G A A G G D A L V A P A * L A L L P P P K**  **9.a. acggcggttagcgacgatgc--gggcggtcagcgacgaggcggccgccatagcggatggcgagaaggcgctgggcgataaggagctgctggaggagacgctctcgtcgcccccgcttaactcgccctccttccgccaccaaaag**  **T A  V  S  D  D  A   G G Q R R G G R H S G W R E G A G R * G A A G G D A L V A P A * L A L L P P P K**  **9.b. acggcggttagcgacgatg------cggccgccatagcggatggcgagaaggcgctgggcgataaggagctgctggaggagacgctctcgtcgcccccgcttaactcgccctccttccgccaccaaaag**  **T A  V  S  D  D  A   A  A  I  A  D  G  E  K  A  L  G  D K  E  L  L  E  E  T  L  S  S  P  P  L  N  S  P  S  F  R  H Q  K**  **10.a. acggcggttagcgacgatgcc------gccgccatagcggatggcgagaaggcgctgggcgataaggagctgctggaggagacgctctcgtcgcccccgcttaactcgccctccttccgccaccaaaag**  **T A  V  S  D  D  A     A  A  I  A  D  G  E  K  A  L  G  D K  E  L  L  E  E  T  L  S  S  P  P  L  N  S  P  S  F  R  H Q  K**  **10.b. acggcggttagcgacgat------------agccatagcggatggcgagaaggcgctgggcgataaggagctgctggaggagacgctctcgtcgcccccgcttaactcgccctccttccgccaccaaaag**  **T A  V  S  D  D   S H S G W R E G A G R * G A A G G D A L V A P A * L A L L P P P K** | **I**  **O**  **I**  **O**  **I**  **O**  **I**  **O**  **O**  **I**  **I**  **O** |
|  | **Black-eye/ CFP^+^**  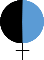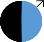 | **1-4. acggcggttagcgacgatg------cggccgccatagcggatggcgagaaggcgctgggcgataaggagctgctggaggagacgctctcgtcgcccccgcttaactcgccctccttccgccaccaaaag**  **T A  V  S  D  D  A   A  A  I  A  D  G  E  K  A  L  G  D K  E  L  L  E  E  T  L  S  S  P  P  L  N  S  P  S  F  R  H Q  K**  **5-9. acggcggttagcgac------gaggcggccgccatagcggatggcgagaaggcgctgggcgataaggagctgctggaggagacgctctcgtcgcccccgcttaactcgccctccttccgccaccaaaag**  **T A  V  S  D    E  A  A  A  I  A  D  G  E  K  A  L  G  D K  E  L  L  E  E  T  L  S  S  P  P  L  N  S  P  S  F  R  H Q  K** | **I**  **I** |
|  | **Tear/CFP^-^**  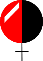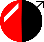 | **1.a. acggcggttagcgacgatgccataggcggcagaggcggccgccatagcggatggcgagaaggcgctgggcgataaggagctgctggaggagacgctctcgtcgcccccgcttaactcgccctccttccgccaccaaaag**  **T A  V  S  D  D  A  I G G R G G R H S G W R E G A G R * G A A G G D A L V A P A * L A L L P P P K**  **1.b. acggcggttagcgacgactgtactgggtgtagtacgggtcgcccggaccgagcggcacgggaaagcactcgggatgttgcggctgccccggatcgcagcactcgatcggcttcccgtccacgccctccttcgagc-----gaggcggccgcc**  **T A  V  S  D  D C T G C S T G R P D R A A R E S T R D V A A A P D R S T R S A S R P R P P S S E A A A** | **O**  **I** |
|  | **Tear/CFP^+^**  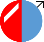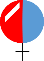 | **1. acggcggttagcgacgatgccataggcggcagaggcggccgccatagcggatggcgagaaggcgctgggcgataaggagctgctggaggagacgctctcgtcgcccccgcttaactcgccctccttccgccaccaaaag**  **T A  V  S  D  D  A  I G G R G G R H S G W R E G A G R * G A A G G D A L V A P A * L A L L P P P K** | **O** |
| **The labeling is the same as Table S4 in addition of R, non-functional resistance allele; r, function resistance allele. Eye phenotypes described in Figure 2.** | | | |

| **Table S7. Progeny phenotypes and genotypes from potential NHEJ resistance-allele/AgNosCd-1 homozygote or heteroallelic intercrosses (Figure 2E).** | | | |
| --- | --- | --- | --- |
|  |  | **Genotypes (DNA and amino acid sequences)** |  |
| **Parent pheno/geno** | **Prog pheno** | **WT acggcggttagcgacgatgccaaggcggccgccatagcggatggcgagaaggcgctgggcgataaggagctgctggaggagacgctctcgtcgcccccgcttaactcgccctccttccgccaccaaaag**  **T A  V  S  D  D  A  K  A  A  A  I  A  D  G  E  K  A  L  G  D K  E  L  L  E  E  T  L  S  S  P  P  L  N  S  P  S  F  R  H Q  K** | **F** |
| **red-eye/CFP^-^ males**  **X**  **red-eye/CFP^-^ females**  **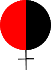**  **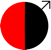**  **R/r or R/R** | **red-eye/CFP^-^**  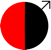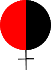 | **1-5.a.acggcggttagcgac------------------------gatggcgagaaggcgctgggcgataaggagctgctggaggagacgctctcgtcgcccccgcttaactcgccctccttccgccaccaaaag**  **T A  V  S  D     D  G  E  K  A  L  G  D K  E  L  L  E  E  T  L  S  S  P  P  L  N  S  P  S  F  R  H Q  K**  **1-5.b.acggcggttagcgacgatgccataggcggcagaggcggccgccatagcggatggcgagaaggcgctgggcgataaggagctgctggaggagacgctctcgtcgcccccgcttaactcgccctccttccgccaccaaaag**  **T A  V  S  D  D  A  I G G R G G R H S G W R E G A G R * G A A G G D A L V A P A * L A L L P P P K** | **I**  **O** |
|  | **black-eye /CFP^-^**  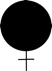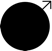 | **1.a. acggcggttagcgacgatgccatcgtcc-------------------------------------------------------------------------------taactcgccctccttccgccaccaaaag**  **T A  V  S  D  D  A I V L T R P P S A T K S**  **1.b. acggcggttagcgacgat---------gccgccatagcggatggcgagaaggcgctgggcgataaggagctgctggaggagacgctctcgtcgcccccgcttaactcgccctccttccgccaccaaaag**  **T A  V  S  D  D    A  A  I  A  D  G  E  K  A  L  G  D K  E  L  L  E  E  T  L  S  S  P  P  L  N  S  P  S  F  R  H Q  K**  **2.a. acggcggttagcgacgatgccataggcggcagaggcggccgccatagcggatggcgagaaggcgctgggcgataaggagctgctggaggagacgctctcgtcgcccccgcttaactcgccctccttccgccaccaaaag**  **T A  V  S  D  D  A  I G G R G G R H S G W R E G A G R * G A A G G D A L V A P A * L A L L P P P K**  **3.a. acggcggttagcgacgatg-----gcggccgccatagcggatggcgagaaggcgctgggcgataaggagctgctggaggagacgctctcgtcgcccccgcttaactcgccctccttccgccaccaaaag**  **T A  V  S  D  D  G G R H S G W R E G A G R * G A A G G D A L V A P A * L A L L P P P K**  **4.a. acggcggttagcgacgatg------------------------gcgagaaggcgctgggcgataaggagctgctggaggagacgctctcgtcgcccccgcttaactcgccctccttccgccaccaaaag**  **T A  V  S  D  D  G    E  K  A  L  G  D K  E  L  L  E  E  T  L  S  S  P  P  L  N  S  P  S  F  R  H Q  K**  **5.a. acggcggttagcgacgatgccataggcggcagaggcggccgccatagcggatggcgagaaggcgctgggcgataaggagctgctggaggagacgctctcgtcgcccccgcttaactcgccctccttccgccaccaaaag**  **T A  V  S  D  D  A  I G G R G G R H S G W R E G A G R * G A A G G D A L V A P A * L A L L P P P K**  **6-8.a.acggcggttagcgacgatgcca-ggcggccgccatagcggatggcgagaaggcgctgggcgataaggagctgctggaggagacgctctcgtcgcccccgcttaactcgccctccttccgccaccaaaag**  **T A  V  S  D  D  A  R R P P * R M A R R R W A I R S C W R R R S R R P R L T R P P S A T K**  **2-8.b.acggcggttagcgacgactgtactgggtgtagtacgggtcgcccggaccgagcggcacgggaaagcactcgggatgttgcggctgccccggatcgcagcactcgatcggcttcccgtccacgccctccttcgagcg-----aggcggccgcc**  **T A  V  S  D  D C T G C S T G R P D R A A R E S T R D V A A A P D R S T R S A S R P R P P S S E A A A**  **2-8.c.acggcggttagcgacgactgtactgggtgtagtacgggtcgcccggaccgagcggcacgggaaagcactcgggatgagatgagtcgccaggggagtggaacgggacggcggttagcgacgatgccatacgcgggag-----aggcggccgcc**  **T A  V  S  D  D C T G C S T G R P D R A A R E S T R D E M S R Q G S G T G R R L A T M P Y A G E A A A** | **O**  **I**  **O**  **O**  **I**  **O**  **O**  **I**  **I** |
| **The labeling is the same as Table S6. Eye phenotypes described in Figure 2.** | | | |

| **Table S8. Sequence verification of the red-eye, CFP^-^, *cd* drive-resistant line Ag*cd*^Δ11,14^.** | | |
| --- | --- | --- |
| **Wild-type** | **acggcggttagcgacgatgccaaggcggccgccatagcggatggcgagaaggcgctgggcgataaggagctgctggaggagacgctctcgtcgcccccgcttaactcgccctccttccgccaccaaaag**  **T A  V  S  D  D  A  K  A  A  A  I  A  D  G  E  K  A  L  G  D K  E  L  L  E  E  T  L  S  S  P  P  L  N  S  P  S  F  R  H Q  K** | **Frame** |
| **Δ11** | **acggcggttagcgacg-----------gccgccatagcggatggcgagaaggcgctgggcgataaggagctgctggaggagacgctctcgtcgcccccgcttaactcgccctccttccgccaccaaaag**  **T A  V  S  D   G R H S G W R E G A G R * G A A G G D A L V A P A * L A L L P P P K** | **Out** |
| **Δ14** | **acggcggttagcg--------------gccgccatagcggatggcgagaaggcgctgggcgataaggagctgctggaggagacgctctcgtcgcccccgcttaactcgccctccttccgccaccaaaag**  **T A  V  S   G R H S G W R E G A G R * G A A G G D A L V A P A * L A L L P P P K** | **Out** |
| Sequences listed in 5’-3’ orientation. Both Δ11 and Δ14 result in out-of-frame reading shifts. Single-letter amino acid codes are aligned with their respective codons. Blue: gRNA sequence; brown: PAM; red: deletion; *: stop codon. | | |

| **Table S9. Phenotypic proportions of progeny of a cross of homozygous resistant line Ag*cd ^Δ11,14^* males to homozygous female AgTP13.** | | |
| --- | --- | --- |
|  | **Phenotypes^1^** | |
| **Generation** | **red-eye (*cd*)/CFP^+^** | **red-eye (*cd*)/CFP^-^** |
| **F1** | **100%**  **(155/155)** | **---** |
| **F2** | **70.7%**  **(549/777)** | **29.3%**  **(228/777)** |
| **F3** | **88.3%**  **(492/557)** | **11.7%**  **(65/557)** |
| **F4** | **82.4%**  **(661/802)** | **17.6%**  **(141/802)** |
| **F5** | **70.3%**  **(248/353)** | **29.7%**  **(105/353)** |
| **F6** | **78.8%**  **(413/524)** | **21.2%**  **(111/524)** |
| **F7** | **76.1%**  **(686/902)** | **23.9%**  **(216/902)** |
| **F8** | **81.1%**  **(271/334)** | **18.9%**  **(63/334)** |
| ^1^All parents and progeny have the red-eye (*cd*) phenotype. The percentages of the phenotypes and number of individuals are listed. The blue- and un-shaded columns are CFP^+^ and CFP^-^ progeny, respectively. | | |

| **Table S10. Phenotypic proportions of progeny of crosses of male homozygous resistant line Ag*cd^Δ11^* to female X1-WT.** | | | | | | | | |
| --- | --- | --- | --- | --- | --- | --- | --- | --- |
|  | **Phenotypes** | | | | | | | |
| **Generation** | **black-eye/CFP^-^** | | | | **red-eye (*cd*)/CFP^-^** | | | |
|  | **Rep 1** | **Rep 2** | **Rep 3** | **Total^1^** | **Rep 1** | **Rep 2** | **Rep 3** | **Total^1^** |
| **F1** | **288** | **492** | **382** | **1162**  **(100%)** | **0** | **0** | **0** | **0**  **(0%)** |
| **F2** | **189** | **209** | **307** | **705**  **(66.3%)** | **108** | **141** | **109** | **358**  **(33.7%)** |
| **F3** | **422** | **234** | **370** | **1026**  **(74.7%)** | **167** | **84** | **96** | **347**  **(25.3%)** |
| **F4** | **223** | **241** | **402** | **866**  **(74.8%)** | **77** | **98** | **117** | **292**  **(25.2%)** |
| **F5** | **328** | **367** | **483** | **1178**  **(74.7%)** | **118** | **113** | **167** | **398**  **(25.2%)** |
| **F6** | **147** | **186** | **147** | **480**  **(68.8%)** | **69** | **86** | **63** | **218**  **(31.2%** |
| **F7** | **137** | **103** | **159** | **399**  **(48.2%)** | **142** | **140** | **147** | **429**  **(51.8%)** |
| **F8** | **205** | **194** | **196** | **598**  **(58.8%)** | **158** | **161** | **98** | **417**  **(41.2%)** |
| **F9** | **283** | **226** | **172** | **681**  **(57.1%)** | **90** | **234** | **187** | **511**  **(42.9%)** |
| **F10** | **279** | **132** | **194** | **605**  **(60.9%)** | **57** | **167** | **165** | **389**  **(39.1%)** |
| ^1^ The total and percentage of each phenotype counted in each population are listed.  Replicates (Reps) start with 150 Ag*cd^Δ11^* males crossed with 150 X1-WT females. The gray- and red-shaded columns are black-eye and red-eye (*cd*) progeny, respectively. | | | | | | | | |


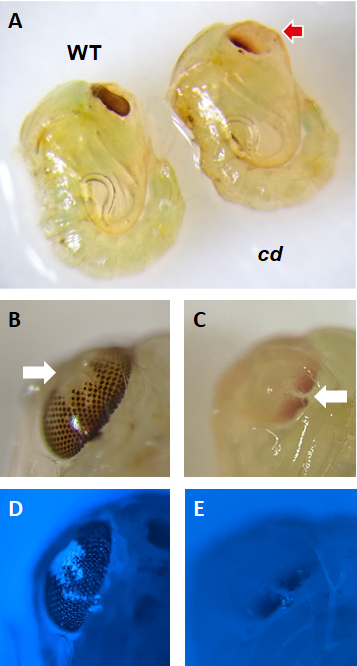


**Figure S1. Mosaic (tear) phenotypes in pupae.** The mosaic phenotype can be seen in both pupal and adult eyes. (A) Non-mosaic phenotypes, black-eye (wild-type, WT) and red-eye (*cd*) (red arrow). Mosaic phenotypes (white arrows) in (B) black-eye (wild-type, WT) and (C) red-eye (*cd*) background The former is positive (D) and the latter negative (E) for CFP fluorescence. Images courtesy of Kiona Parker.
